# Supplementary material for: Moulding hydrodynamic 2D-crystals upon parametric Faraday waves in shear-functionalized water surfaces
Source: Nat Commun. 2021 Feb 18;12:1130. doi: 10.1038/s41467-021-21403-0 (PMC7892544; doi:10.1038/s41467-021-21403-0)
Supplement: Supplementary file 1 — Supplementary Information [file 41467_2021_21403_MOESM1_ESM.pdf]

Supplementary Material for

Moulding hydrodynamic 2D-crystals upon parametric Faraday waves in shear-functionalized water surfaces

Mikheil Kharbedia[1], Niccolò Caselli[1,2], Diego Herráez-Aguilar[3],
Horacio López-Menéndez[1,2], Eduardo Enciso[1], José A. Santiago[1,4]
and Francisco Monroy[1,2,*]

[1]Department of Physical Chemistry, Universidad Complutense de Madrid, Ciudad Universitaria s/n E28040 Madrid (Spain).

[2]Unit of Translational Biophysics, Instituto de Investigación Sanitaria Hospital Doce de Octubre, E28041 Madrid (Spain).

[3] Instituto de Investigaciones Biosanitarias, Universidad Francisco de Vitoria, Ctra. Pozuelo-Majadahonda, 28223, Pozuelo de Alarcón, Madrid (Spain).

[4] Matemáticas Aplicadas y Sistemas, Universidad Autónoma Metropolitana Cuajimalpa, Vasco de Quiroga 4871, 05348 Ciudad de México (México).

***e-mail:** monroy@quim.ucm.es

Table of Contents

Supplementary Figures 1-10

Supplementary Tables 1-2

Supplementary Notes 1-6

Supplementary Figures

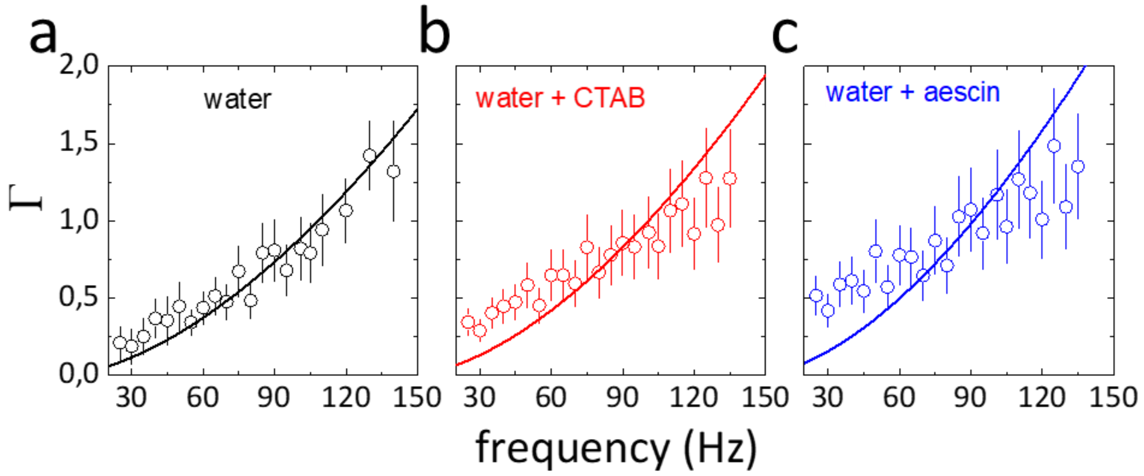

Supplementary Figure 1. Onset of the Faraday instability for the bare water surface and functionalized with adsorbed monolayers. The critical acceleration $\Gamma = a/g$ was measured as a function of excitation frequency for Faraday waves in pure water (a), and in aqueous solutions of CTAB 1.5 mM (b) and aescin 1.0 mM (c). Both concentrations are above the respective cmc. The circles correspond to experimental data determined as the onset of the Faraday instability observed at increasing excitation amplitude (the average values correspond to at least five independent measures with error bars corresponding to the standard deviation). The solid curves correspond to Eq. (1) of the main manuscript, which reproduces the expectation for the critical acceleration to induce Faraday waves restored by surface tension (water: $\sigma = 72\, mN/m$, CTAB: $\sigma = 50\, mN/m$, aescin: $\sigma = 30\, mN/m$,) in a bulky liquid enclosed by a vibrating receptacle ($\rho = 10^{-3} kg\; m^{-3}$ and $\eta = 10^{-3} Pa\; s$). Note that the data agree with theoretical predictions, although in the case of water surface covered by aescin a small deviation at low frequency is found.

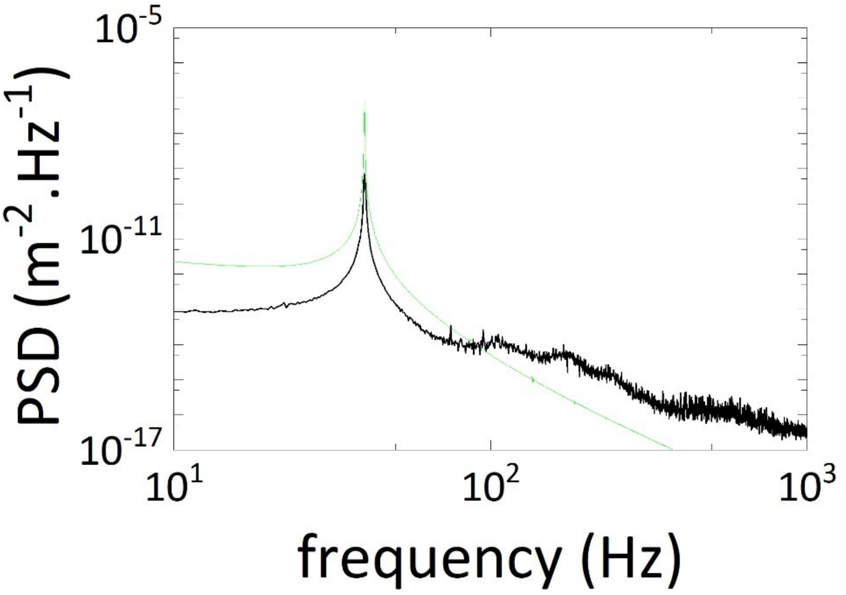

Supplementary Figure 2. Linear response at low driving amplitudes. Surface wave spectrum in the linear response regime (black curve), obtained for very low vertical excitation amplitude $\Gamma = 0.03 \ll \Gamma_F$, at $\omega_0 = 47$ Hz (capillary regime).The observed resonance occurs exactly at the pumping frequency induced by the lauder (green curve). The observed spectral broadening is compatible with the natural bandwidth of the excitation channel (green straight line).

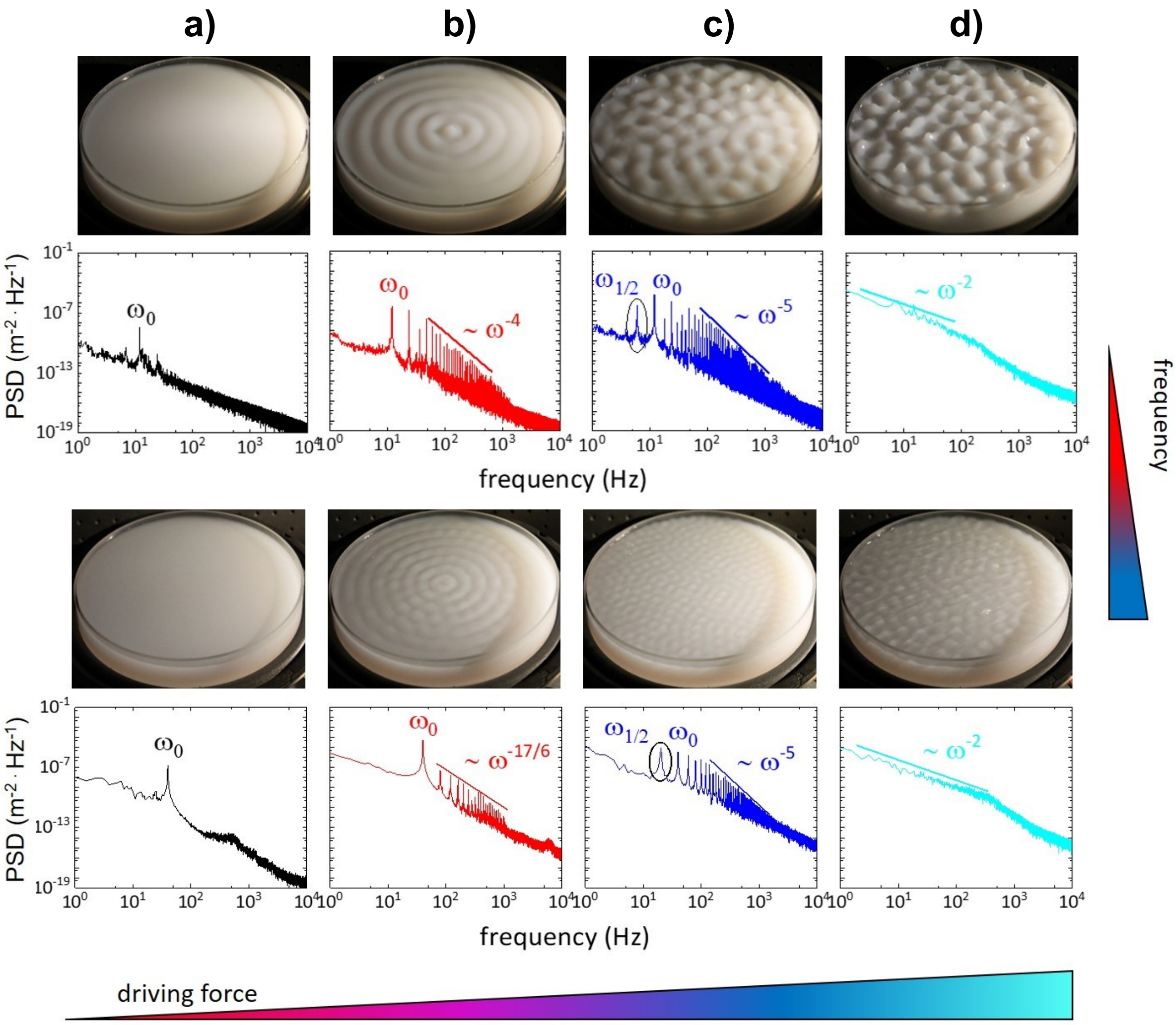

Supplementary Figure 3. Nonlinear surface waves regimes in water surface. Images and spectral response of nonlinear surface waves driven on pure water under parametric excitation ($\omega_0 = 12Hz$ and $\omega_0 = 47Hz$, upper and down panels, respectively). For increasing vertical acceleration determining the driving force (from a to d) different wave regimes were observed, as described in the main paper: (a) linear regime exhibiting a single peak at ω_0, (b) Kolmogorov-Zakharov spectrum of weak wave turbulence exhibiting container-shape surface waves and a $\omega^{-17/6}$-intensity decay typical of cascades of nonlinear capillary waves, (c) Faraday regime exhibiting disordered surface roughening and a spectrum with subharmonic response at $\omega_0/2$, and an intensity decay cascade that scales as ω^{-5}; (d) Chaotic regime featuring strongly disordered surface and a continuous spectrum (without any resonance) and a intensity decay proportional to ω^{-2}.

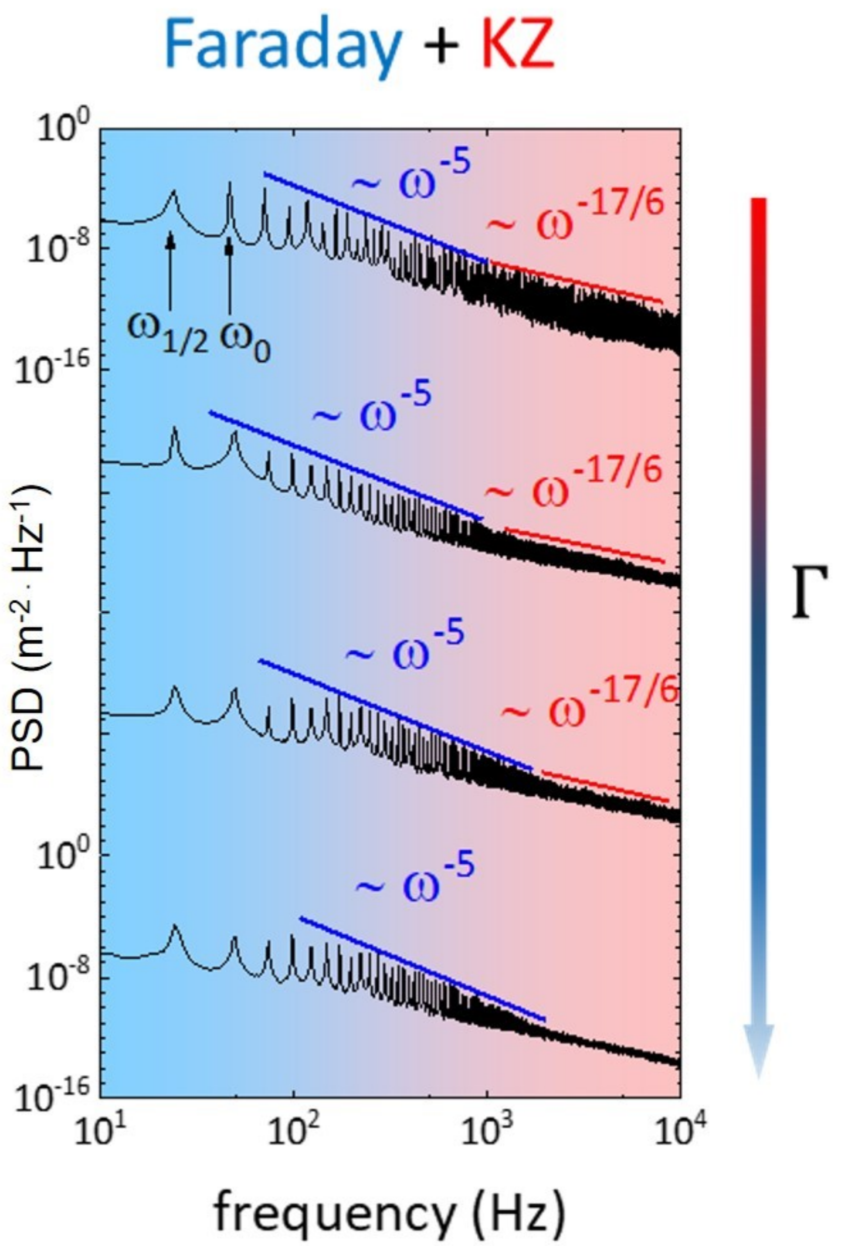

Supplementary Figure 4. Hybrid spectrum of turbulence for pure water in the capillary regime ($\omega_0 = 47Hz$) where both Faraday (F: $\sim\omega^{-5}$) and Kolmogorov-Zakharov (KZ: $\sim\omega^{-17/6}$) spectral decay coexist. The former was found in the low frequency range, while the latter in the high frequency spectrum. For increasing $\Gamma\,(0.07-2.5)$ the hybrid spectrum converts into a pure Faraday wave cascade. As discussed in the main manuscript, the KZ spectrum is restricted to the higher frequencies range due to its lower intensity in comparison with Faraday wave cascade.

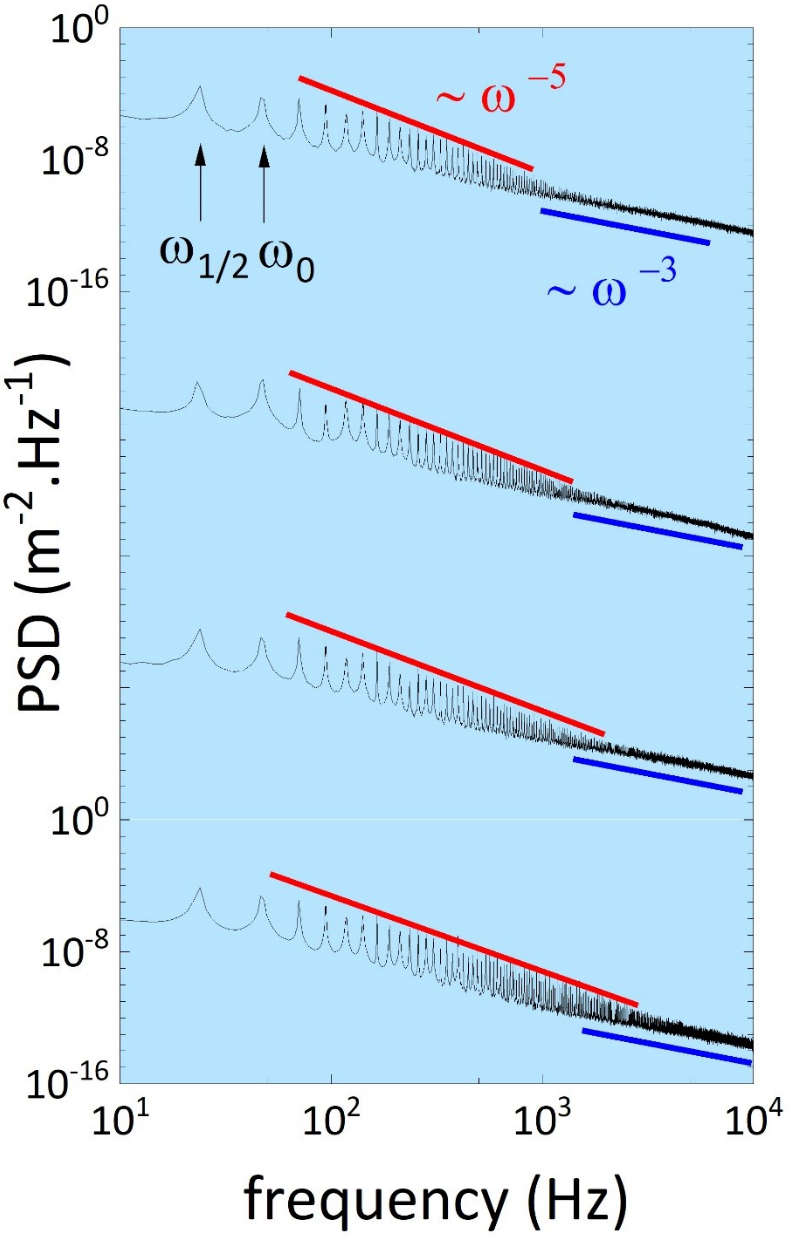

Supplementary Figure 5. Pure Faraday wave spectra (with intensity decay $\sim \omega^{-5}$) in the capillary regime ($\omega_0 = 47Hz$) for bare water surface, depicted in the Γ range $(0.3 - 0.7)$ to avoid hybrid spectrum of turbulence. The pure-FW envelope decay of the developed modes ($\sim \omega^{-5}$) contends with the frictional background dominant at high frequencies ($\sim \omega^{-3}$). The cascades are composed by harmonics $\omega_n = n\omega_0$, and subharmonics $\omega_{n/2} = n\omega_{1/2}$. This physics compatible with resonance between the liquid inertia and the external driving force is explained in Supplementary Note 1.

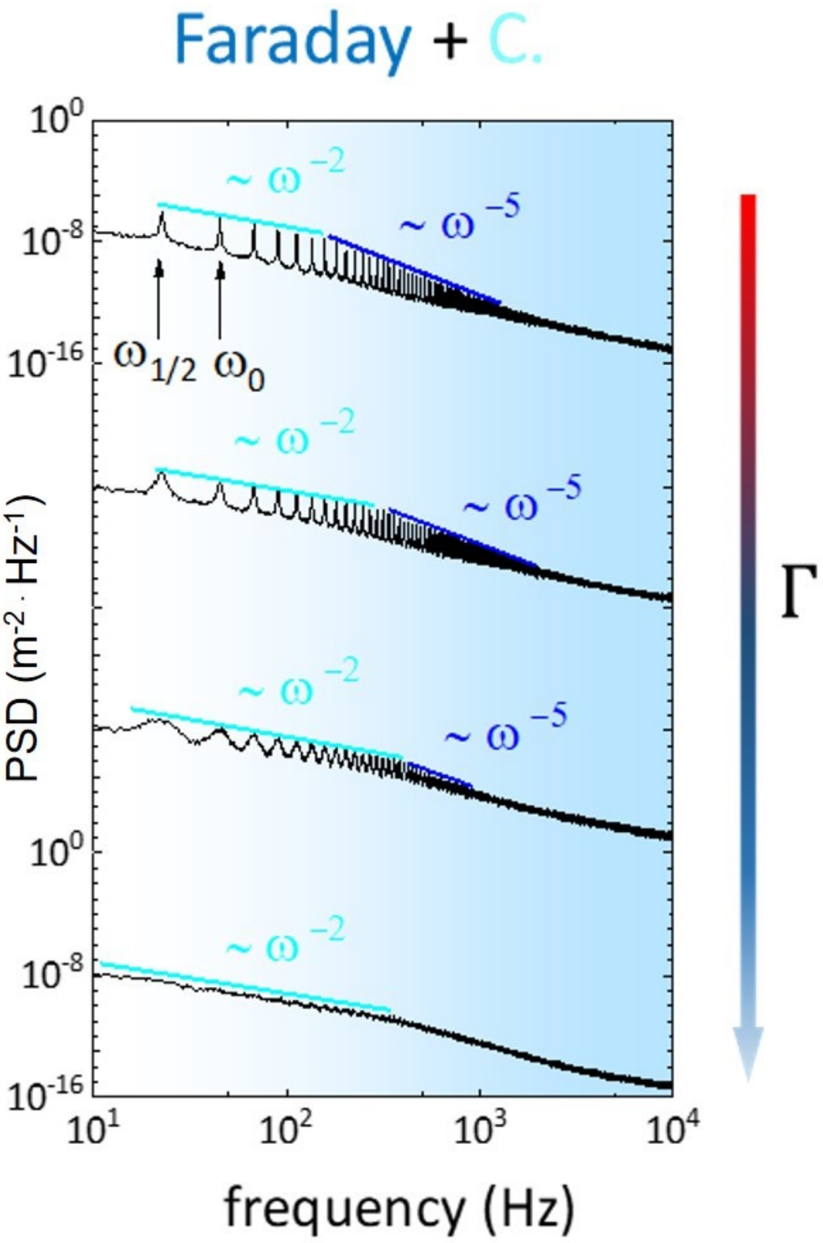

Supplementary Figure 6. Hybrid spectrum of turbulence obtained at the transition from Faraday to continuous spectrum for bare water surface in the capillary regime ($\omega_0 = 47Hz$) by increasing Γ in the range $(0.8 - 2)$. At higher values of Γ continuous spectrum emerges with characteristic Brownian-like energy cascade ($\sim\omega^{-2}$). This cascade firstly develops in the lower frequency range and, for increasing Γ, propagates to higher harmonics. Also, it is noteworthy the fact that for increasing excitation amplitudes the spectral peak broad until they disappear at higher Γ.

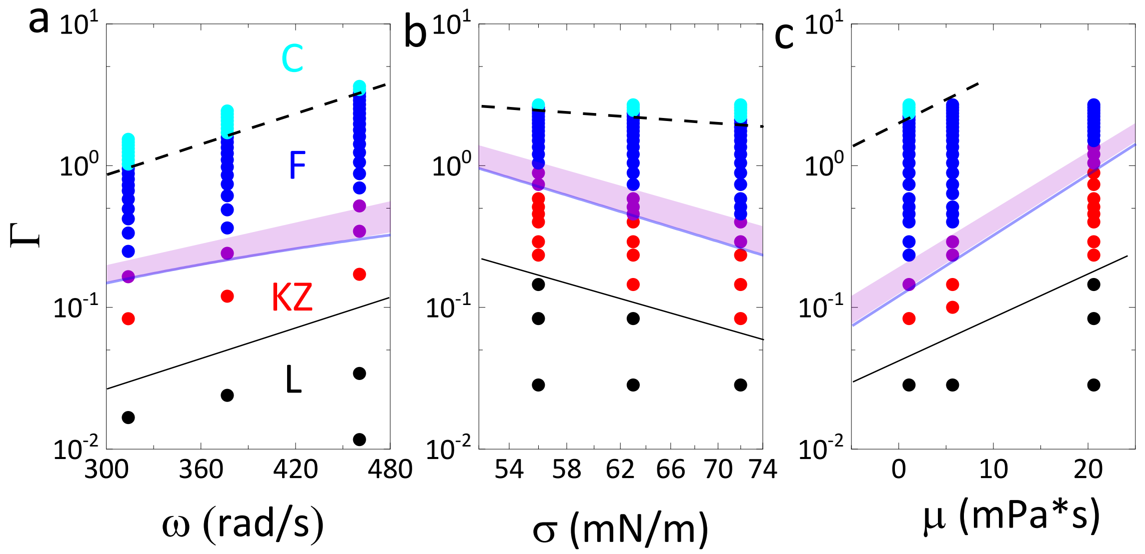

Supplementary Figure 7. NLSW-states in liquid surfaces of variable surface tension and viscosity. a) State diagram of nonlinear surface waves in water under parametric excitation, as a function of excitation frequency ω_0 and the reduced acceleration Γ. b) State diagram for different surface tension σ, varied by increasing CTAB concentration close to its cmc. c) State diagram for different liquid viscosity μ, changed by increasing the glycerol concentration in water. In each of the three presented scenarios, five different states are observed: i.e. linear (black dots), KZ (red dots), hybrid KZ-Faraday (pink band), pure Faraday (blue dots) and continuous spectrum (light blue dots). The blue solid line corresponds to Eq. (1) of the main manuscript, which coincides with the KZ to Faraday spectrum transition. The black dashed line indicates the experimental transition from Faraday to continuous spectrum. The black solid line the transition from linear to KZ spectrum. Finally, the pink area represents the hybrid Faraday-KZ spectral region.

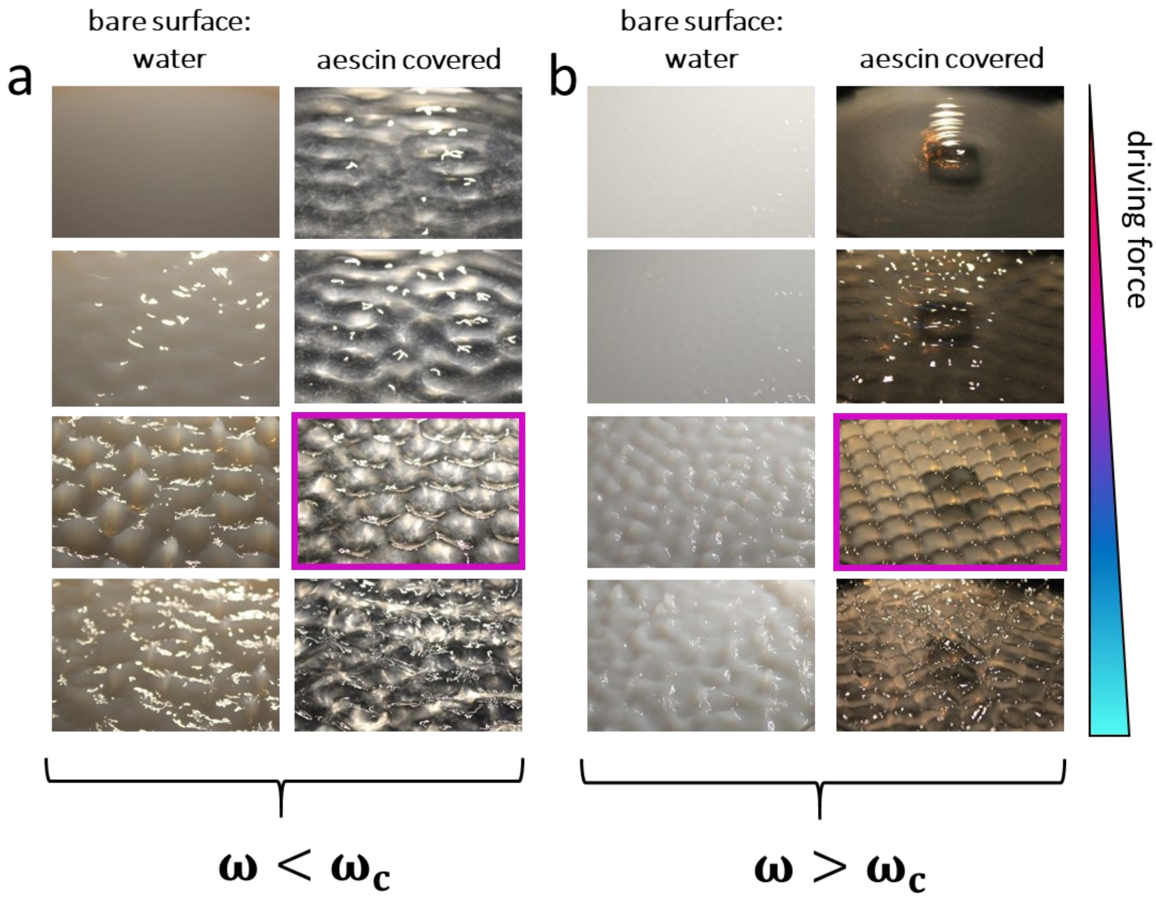

Supplementary Figure 8. a) Comparison of images of bare water (left panels) and aescin covered (right panels) surface waves driven in the gravity regime ($\omega_0 = 10Hz$) for increasing vertical acceleration (from top to bottom panels). The aescin concentration was 1 mM. Only the introduction of aescin was able to induce an ordered surface pattern on the Faraday waves (magenta surrounded box) b) Comparison of images of bare water (left panels) and aescin covered (right panels) surface waves driven in the in the capillary regime ($\omega_0 = 47Hz$) by increasing the vessel vertical acceleration (from top to bottom panels). By increasing the vertical acceleration, surfaces waves emerged due to the parametric resonance and organized in a regular packing leading to the formation of a hydrodynamic crystals which achieved their maximum packing order at intermediate accelerations (magenta surrounded boxes). By further increasing the driving acceleration a crystal breakup occurred.

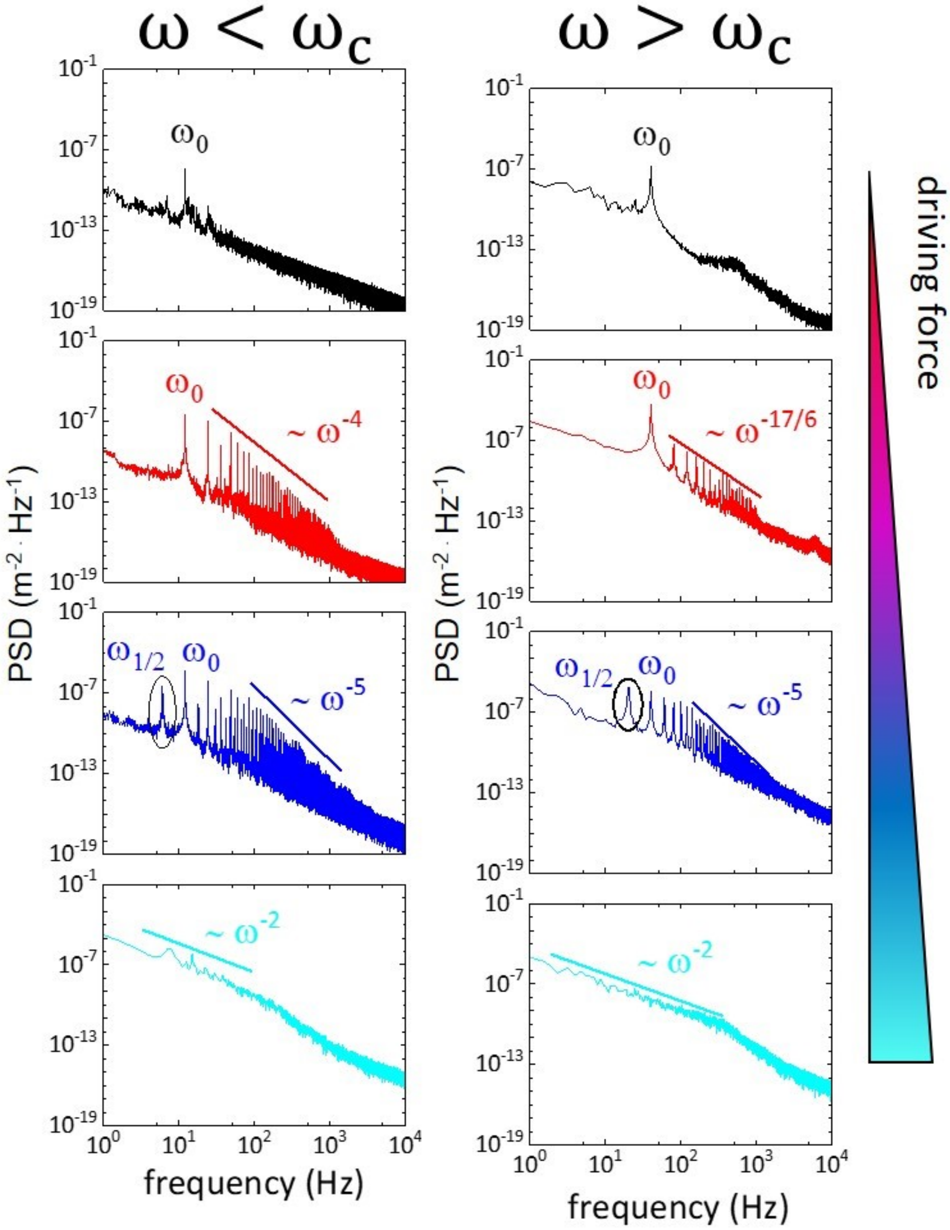

Supplementary Figure 9. Representative log-log spectra of nonlinear surface waves turbulence in aescin covered water surface. Below the capillary frequency gravity waves (left panels) are found, while capillary waves (right panels) are observed above. The sequence of spectra spans from top with the linear regime (only the fundamental response is present at ω_0) to bottom; then, the incoherent KZ-turbulence characterized by a spectral decay exponent α that depends on the dispersion regime ($\alpha = -4$ for gravity regime and $\alpha = -17/6$ for capillary regime), through of the Faraday instability (where the subharmonic response appears at $\omega_{1/2} = \omega_0/2$ and the Faraday waves turbulent cascades decay as $\alpha = -5$), up to the chaotic regime in the lower panels ($\alpha = -2$). Crystal formation is only observed in the Faraday regime ($\alpha = -5$).

Supplementary Tables

Supplementary Table 1

Critical acceleration thresholds for the non-linear surface-wave states

We considered bare water surface with a high surface tension, low-tension aqueous surface with a CTAB-adsorption layer (light gray), and high-viscosity water/glycerol mixture (dark gray). We report the experimental values of critical acceleration (a_X, where X stands for L: linear; KZ: Kolmogorov-Zakharov; F: Faraday; C: Chaotic) on the onset of different parametric wave states as quantitatively identified by the slope of the turbulence cascade detected by means of laser Doppler vibrometry (LDV). The onset for Faraday instability (a_F; marked in blue) measured as a function of the driving frequency (ω_0) follows the theoretical prediction as given by Eq. (1) of the main text for the quoted values of surface tension (σ) and the kinematic viscosity (η) (see Supplementary Figure 1 and Fig. 3c and the main text for details). The Faraday wave (FW)-dispersion status is determined by the value of ω_0 with respect to the capillary frequency ω_c (for water, $\omega_c = 14\,Hz$). Thus, gravity waves occur below or close or below ω_c, whilst capillary waves emerge for $\omega_0 > \omega_c$. To study the FWs in a defined dispersion regime, the experiments reported in the main manuscript corresponded to the reference frequency $\omega_{ref} = 47Hz$, i.e. in the capillary regime. By analysing the results reported in this Supplementary Table 1, the dominance of inertia in creating NLSWs is evident, since stronger forces are needed with decreasing surface tension (in CTAB solutions) and increasing viscosity (in water/glycerol mixtures).

Liquid surface	ω_0 (Hz)	σ (mN/m)	η (mPa.s)	a_L (m/s^2)	a_{KZ} (m/s^2)	a_F (m/s^2)	a_c (m/s^2)
water ($\omega_0 < \omega_c$)	10	72	1	0.0001	0.0005	0.001	0.003
water ($\omega_0 \approx \omega_c$)	15	72	1	0.0002	0.0006	0.002	0.006
water ($\omega_0 > \omega_c$)	25	72	1	0.0004	0.001	0.006	0.03
water ($\omega_0 \gg \omega_c$)	35	72	1	0.001	0.03	0.01	0.6
water ($\omega_0 = \omega_{ref}$)	47	72	1	0.01	0.8	2	10
water + 0.1mM CTAB	47	62	1	0.02	1.2	4.1	22
water + 1mM CTAB	47	55	1	0.02	1.9	6.7	25
water ($\omega_0 > \omega_{ref}$)	60	72	1	0.01	1.1	2.3	15
water ($\omega_0 \gg \omega_{ref}$)	73	72	1	0.01	1.6	3	25
water + 5% glycerol	47	72	6	0.2	1	2	>30
water + 15% glycerol	47	72	28	0.2	2.3	9	>30

Supplementary Table 2

Critical acceleration thresholds in water covered with aescin

To study the appearance of NLSWs in the different regimes of wave propagation, we focused on the amplitude transitions at variable acceleration in three well-delimited dispersion regimes; these are: hybrid gravity-capillary waves close to the capillary frequency (GCW at $\omega \approx \omega_c = 14\,Hz$), pure gravity waves (GW at $\omega \ll \omega_c$) and pure capillary waves (CW at $\omega \gg \omega_c$). We detected the acceleration thresholds as reported in Supplementary Table 1 (the onset of the Faraday instability is marked in blue). No significant differences were detected in comparison with the bare surface of water.

ω_0 (Hz)	dispersion	σ (mN/m)	η (mPa s)	a_L (m/s^2)	a_{KZ} (m/s^2)	a_F (m/s^2)	a_c (m/s^2)
10	GW	40	1	0.0001	0.0003	0.001	0.005
15	CGW	40	1	0.0002	0.005	0.02	0.04
25	CGW	40	1	0.005	0.01	0.04	0.07
35	CW	40	1	0.01	0.16	0.5	0.6
47	CW	40	1	0.01	0.4	1.6	9
60	CW	40	1	0.05	0.5	3	14
73	CW	40	1	0.1	1	5	25

Supplementary Notes

Supplementary Note 1

Resonance status of nonlinear water surface wavefields: hydrodynamic regimes and spectral signatures

1-1. Nonlinear surface waves: resonant interactions
1-2. Nonlinear stiffening as a NLSW organizer
1-3. Towards a minimal NLSW theory
1-4. Mechanical equivalent: Duffing nonlinear oscillator
1-5. Faraday waves as a parametric resonator
1-6. Power spectral density
1-7. Model prediction: DNLO spectral slopes
1-8. DNLO harmonic balance: inertial and frictional signatures
1-9. Inertial FW domain: resonant mass-force spectrum
1-10. Loosing Faraday wavefield self-focussing: frictional death
1-11. Transition to unsteady chaotic regime: Landau's spectrum
1-12. DNLO chaotic regime: Landau's mode superposition

1-1. Nonlinear surface waves: resonant interactions

The nonlinear hydrodynamics of interacting water waves has been the subject of intensive research as a first principle of fluid motion in two dimensions [1-3]. The notion of resonant interaction has been central on all those advances not only as constitutes the hydrodynamic skeleton for harmonic ensembles of nonlinear surface waves (NLSW) [3-5], but specially for building Faraday waves (FWs) at subharmonic resonance [6,7]. Fluid surfaces respond to periodic forces at their natural frequencies as far as they behave as harmonic oscillators when coupled to their bulk counterpart [8]. By forcing a fluid to oscillate, different classes of NLSWs are generated with a hydrodynamic structure that depends on two key factors [5-9]:

a) The specific nature of the driving force; either random or monochromatic, eventually leading harmonic resonance under extrinsic forcing.

b) The underlying hydrodynamic skeleton; inducing wavefield interactions and resonances between coupled surface modes.

As a prevalent conceptualization, the stable NLSW states obtained under different classes of external forcing (stable turbulence, condensates, solitons, vortices, etc.) are conceived as coupled waves with a hierarchical energy distribution determined by the specific structure of the involved resonances, either intrinsic, extrinsic, or both [10-12]. Such mainstream wisdom emphasizes the special role of resonant interactions, so-called because they have the mathematical form of resonantly forced, nonlinear oscillators (NLOs). These resonant interactions can cause significant energy transfer among NLSWs and profoundly affect wavefield evolution. Here, one-dimensional NLO wave equations are introduced as a model for assessing the spectral slopes observed in experiments.

1-2. Nonlinear stiffening as a NLSW-FW organizer

As refers to the new class of surface hydrodynamic skeletonization involved in this work (as a stiffness-induced coherent ordering of the Faraday wavefield at a fluid surface), the ordered NLSWs appeared as FW patterns (hydrodynamic crystals) are envisaged to emerge as ordered condensates of harmonic and subharmonic resonances with the external monochromatic source of driving [5-7].

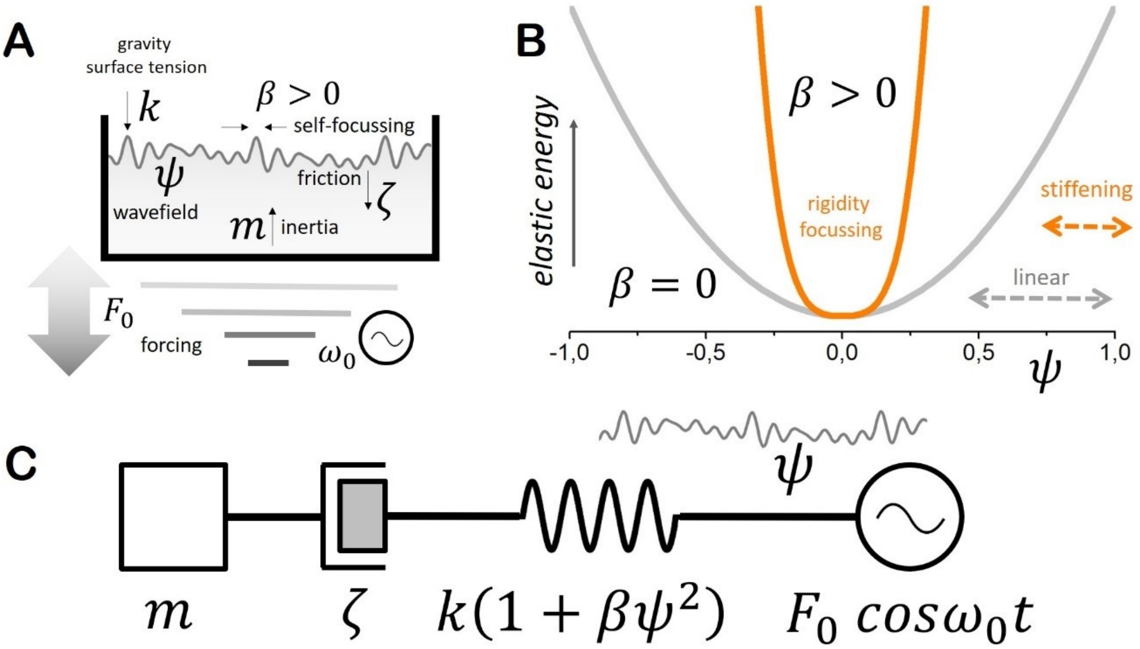

Supplementary Figure N1. Mechanical equivalent of nonlinear surface wave motion in a vertically vibrated liquid. We argue around a minimal dynamical depiction as a Duffing Non-Linear Oscillator (DNLO) that qualitatively explains the main features observed in experiments. A) Schematics on NLSW-DNLO dynamics as a transverse surface wavefield ψ externally excited upon liquid vibration that is globally exerted in the container by an oscillating AC driver (of force amplitude F_0 and monochromatic frequency ω_0). This waving motion is restored by liquid inertia and surface elasticity

(linear and nonlinear), and frictional dissipation operated under viscous drag. Mechanical ingredients*:* effective inertial mass defining linear momentum (m); surface elastic response as a linear spring constant (k; which recapitulates gravity and surface tension); Duffing-like wavefield self-interaction as produced upon nonlinear surface elasticity (β is a nonlinear rigidity). B) Elastic free-energy landscape. A Hookean harmonic response characterized by the linear spring constant is assumed at a first instance. Nonlinear rigidity causes the quadratic symmetry to be broken in the Faraday wave self-focusing characteristic of the hydrodynamic crystals experimentally observed upon surface stiffness functionalization with adsorbing aescin (FW-freezing). The nonlinear coefficient β represents either surface stiffening leading wave self-focussing ($\beta > 0$), or surface softening causing wave defocussing ($\beta < 0$). C) Mechanical equivalent corresponding to the force ingredients depicted in A) as coupled in series; from left to right: inertial mass (m); viscous damper with Newtonian friction coefficient (ζ); elastic spring (k is the Hooke-spring constant; β the nonlinearity rigidity coefficient describing the strength for wavefield self-interaction); time dependent external force $F(t) = F_0 cos(\omega_0 t)$, which excites the DNLO at its natural frequency $\omega_0 = (k/m)^{1/2}$.

The Supplementary Figure N1 depicts a nonlinear oscillator (NLO) implementation emerged from the minimal set of coupled resonances between the systemic response and the external force. The surface-induced shear rigidity plays a structuring role as a wavefield self-focusing interaction, which elicits locked resonances leading the formation of hydrodynamic crystals as free-standing NLSW-FWs with a permanent form. As a consequence of surface stiffening, the FW-patterns should appear upon external driving when the oscillating components of the surface stresses move in congruence with the harmonic NLSWs excited at resonant frequencies [4]. A sort of energy cascade with a rigidity-imprinted signature should be happening within the NLSW-FW pattern.

As a minimal implementation we propose a mechanical model with subharmonic resonances (FW alike) at the core of the field self-focussing interaction (FW-ordering). Supplementary Figure N1 recapitulates the systemic ingredients of our model (see caption for details). The theoretical framework here proposed depicts a unifying platform to settle further predictions (see Supplementary Note N2), which are oriented to future exploitations of the hydrodynamic crystal concept (see Supplementary Note 3).

1-3. Towards a minimal NLSW theory

Harmonic resonance is assumed the organizational principle that guides NLSW ordering in self-focussed wavefields. We invoke the essential ingredients of harmonic resonance to build a mechanical equivalent system at extrinsic resonance with the

driving force. Our theoretical construction captures the emergence of FWs as parametric resonances with liquid inertia, the so-called mass-force resonances. We then suggest hydrodynamic crystals as FW-moulded wavefields with a standing wave pattern coherently imposed by a structure of harmonic (and subharmonic) resonances with the external source. Surface stiffness is assumed to be the key factor leading to enough phase coherence upon wavefield self-focusing. Chaos is ultimately viewed as a frictional dominance of decoherence at high wavefield velocities. The weak turbulence regime is finally envisaged as the natural response at low amplitude when mass-force resonance does not occur yet. In this sense, the classical Kolmogorov-Zakharov (KZ) picture of dispersion-governed weak wave turbulence will be recovered [3,10]. A one-dimensional wavefield $\psi(x,t)$ is assumed as the mechanical response in a single surface point upon external driving with a monochromatic AC structure $F(t) = F_0 cos(\Omega t)$ (of amplitude F_0 and constant frequency Ω; see Fig. SN1A). External forcing drives the natural response at harmonic frequencies $\omega_n = n\omega_0$ (matching the driving frequency; $\omega_0 = \Omega$), and subharmonic frequencies $\omega_{n/2} = n\omega_{1/2}$ (with $\omega_{1/2} = \omega_0/2$ for the FWs [13,14]).

The minimal prerequisite for harmonic resonance is some *spring* system that when deflected from some rest state experiences a restoring force that pushes it back toward the equilibrium state. We assume the surface Hookean response embedded into a spring constant $k(g,\sigma)$, as composed of the gravity and capillary restoring forces; this fixes the natural frequency at $\omega_0 = (k/m)^{1/2}$.

Also required is *bulk inertia*, or momentum ingredient, that makes the wavefield to overshoot the equilibrium point and pass on through. As an indispensable ingredient for FWs, we consider liquid inertia embedded in a fictitious inertial mass m that enables the elastic surface to oscillate between a mass-force system that allows parametric resonance as a time-dependent restoring force. The system is driven back and forth the equilibrium at frequency $\omega_{1/2}$ [5,8]. Bulk inertia is usually neglected in the models of weak NLSW [3,9-12], which focus on statistical kinetic equations for the random wavefield velocities as governed by interactions between the dispersive surface modes (see Supplementary Figure 1). The FW regime has been shown as dominated by bulk inertia [13-16], exhibiting a very characteristic spectral density decay, at ω^{-5} rate, independently of the wave dispersion domain (see Supplementary Figure 3).

Furthermore, bulk friction is considered as a sink for dissipation. The viscous drag ingredient is described by a friction coefficient ζ. We expect a leading role for friction in decohering resonant correlations at increasing velocity, thus being necessary to describe frictional death at low forcing (weak turbulence), and the dynamic pathway to chaos observed at high forcing (Landau's catastrophe).

Finally, as the distinctive component representing stiffening nonlinearity, we consider a self-interacting wavefield term imposed by surface shear rigidity (promoting wave freezing) [15-17]. In a minimal symmetry-preserved, nonlinear form, the wavefield response would be perturbed as $\psi(1 + \beta\psi^2 + \cdots)$, with β being a stiffening constant leading to field self-focussing (see Supplementary Figure N1 B). The stiffening coefficient represents the real surface shear rigidity (G, as imparted by the functionalizing agent aescin), which entails a stiffer elasticity well than the Hookean quadratic well. A stiffening universalization (dominance of surface rigidity over capillarity or gravity) is expected for NLSWs in rigid surfaces at sufficiently high nonlinearity, i.e. when $\beta\psi^2 \gg 1$ for $\beta(G) > 0$ (Supplementary Figure N1 B). As a matter of conjecture, this ingredient would capture wavefield self-focussing as the key factor for the phase locking required for stable FW patterning (see Figure 4).

1-4. Mechanical equivalent: Duffing nonlinear oscillator

We consider the vertical displacement at a single surface point (see Supplementary Figure N1). The wavefield is folded in $\psi(x,t)$ as captures a discretized NLSW ensemble $\psi = \{\psi_n\}$, which is extrinsically determined under a highly narrow distribution of normal stresses driven by the monochromatic source. Because the injected energy is assumed to continuously flow from the driving source through the vibrating liquid up to the surface (where it spreads over the whole NLSW ensemble), we expect the above dynamical components as coupled in series. Once the fluctuating parts have been separated from the mean flow, our deterministic description of the wavefield adopts the form as a dynamical system with the mathematical formulation of a resonantly forced, nonlinear oscillator (NLO) [8,15,16]; this is written as the Duffing oscillator in a generalized form:

$$m\ddot{\psi} + \zeta\dot{\psi} + k\psi(1 + \beta\psi^2) = F(t) \qquad (1)$$

upon a surface response of strength $k(g,\sigma)$, and stiffness $\beta(G)$, at the accessible harmonics ($\omega_n = n\omega_n$), and subharmonics ($\omega_{n/2} = n\omega_{1/2}$). Hereinafter, we will refer to the Duffing-like nonlinear oscillator (aka DNLO generator, or simply DNLO).

Except for the frictional term, the DNLO preserves systemic time-reversal symmetry, a property necessary to sustain a stationary response under oscillatory forcing. The presence of friction causes time-symmetry to break into a direct cascade that pumps the energy from the upper time scale of the driving frequency up to higher frequencies corresponding to smaller scales. The DNLO generates the surface-equivalent wavefield ψ with the differential features discriminated in experiments. For Faraday wavefields, particularly, we describe a dominance of extrinsic mass-force resonance, and its spectral signature, in the corresponding scaling-law decaying cascades of power (with the observed subharmonic response at $\omega_{1/2}$, and the $\alpha = -5$ decay rate of the power spectral densities; see Figure 2).

1-5. Faraday waves as a parametric resonator

Parametric resonance is a phenomenon caused by periodic changes in the spring parameters [15,17]. If the frequency of this modulation is an integer multiple of the natural frequency ω_0, a subharmonic resonance can occur at doubled period ($\omega_{1/2} = \omega_0/2$), causing the newly created mode to become amplified [17]. Faraday waves (FWs) constitute a paradigm of subharmonic NLSW resonance at extrinsic coupling with an external driving force causing vertical liquid vibration [6,2,17]. Because energy is cyclically injected, the FW response has been described as a forced oscillator at parametric resonance [18]. Resonantly forced oscillators like the DNLO, undergo a bifurcated response leading inertia-restored subharmonic response in addition to nonlinear resonance [19]. In quantitative terms, DNLO solutions split in two superposed cascades of harmonics and their counterpart subharmonics as $\psi = \psi_0 + \epsilon\psi_{1/2}$ (likewise for the hybrid KZF-cascades observed in experiments; see Figure 2 in the main text, and Supplementary Figures 3-5). By searching hybrid solutions to Eq. (1), one gets:

$$m(\ddot{\psi}_0 + \epsilon\ddot{\psi}_{1/2}) + \zeta(\dot{\psi}_0 + \epsilon\dot{\psi}_{1/2}) + k(\psi_0 + \epsilon\psi_{1/2})\left[1 + \beta(\psi_0 + \epsilon\psi_{1/2})^2\right] =$$
$$= F(t;\Omega) \qquad (2)$$

Assuming the harmonic cascade dominate over the subharmonics ($\epsilon < 1$), the stiffening Duffing term can be expanded as (up to second leading order):

$$1 + \beta\left(\psi_0 + \epsilon\psi_{1/2}\right)^2 \approx 1 + \beta\psi_0^2 + 2\beta\epsilon\psi_0\psi_{1/2} + \cdots \qquad (3)$$

so that, the time-dependent spring term writes as (up to second perturbative order):

$$\left(\psi_0 + \epsilon\psi_{1/2}\right)\left[1 + \beta\left(\psi_0 + \epsilon\psi_{1/2}\right)^2\right] \approx \left(\psi_0 + \epsilon\psi_{1/2}\right)\left[1 + \beta\psi_0^2 + 2\beta\epsilon\psi_0\psi_{1/2} + o(\epsilon^2)\right] \approx \psi_0(1 + \beta\psi_0^2) + \epsilon\psi_{1/2}(1 + 3\beta\psi_0^2) + o(\epsilon^2), \qquad (4)$$

which is composed of main wavefield nonlinear contributions (up to Duffing-like ψ_0^3), purely linear subharmonic response ($\sim \psi_{1/2}$), and nonlinear coupling terms (involving third-order $\psi_{1/2}\psi_0^2$ interactions accounting for harmonic to subharmonic energy leakage); if second order terms $o(\epsilon^2)$ are drop, Eq. (4) splits in two independent equations describing two wavefields superposed:

1) The main, generating wavefield ψ_0 composed by the fundamental mode (and their harmonics) as excited by the external source:

$$m\ddot{\psi}_0 + \zeta\dot{\psi}_0 + k(1 + \beta\psi_0^2)\psi_0 = F(t;\Omega) \qquad (5)$$

Under oscillatory forcing, it exhibits a natural response at extrinsic resonance with the external source; so that, the fundamental frequency is $\omega_0 = \sqrt{k/m}\,(= \Omega)$. Because of the self-focusing structure of the Duffing-like nonlinearity chosen (cubic spring of elastic restoring force $F_{elast} = -k\psi_0(1 + \beta\psi_0^2)$), only odd harmonics are generated, i.e. $\omega_0, 3\omega_0, 5\omega_0, \ldots$ A complete sequence including even harmonics can be predicted from more complex NLO structures (from possible symmetry breakings, e.g. $F_{elast} = -k\psi_0(1 + \alpha\psi_0 + \beta\psi_0^2)$). For the sake of simplicity, we restrict to the minimal DNLO construction for the FWs:

2) The subharmonic response at parametric resonance $\psi_{1/2}$,

$$\ddot{\psi}_{1/2} + (\zeta/m)\dot{\psi}_{1/2} + \omega_0^2[1 + \epsilon(t)]\psi_{1/2} = 0 \qquad (6)$$

with time-dependent modulation of the spring parameter imposed with the same self-focusing structure as the nonlinear Duffing term [8,17]; this is:

$$\epsilon(t) = 3\beta\psi_0^2(t). \qquad (7)$$

Assuming a parametrically resonant response imposed by the driving force, with leading term in the form of $\psi_0 = A_0 cos(\Omega t)$, Eq. (6-7) correspond the damped Mathieu equation usually invoked to describe the FW subharmonic instability [13,14]:

$$\ddot{\psi}_{1/2} + (\zeta/m)\dot{\psi}_{1/2} + \omega_\Omega^2[p + 2q\, cos(2\Omega t)]\psi_{1/2} = 0 \quad (8)$$

with a natural response at resonant frequencies ω_Ω's, and Mathieu parameters:

$$p = 1 + 2q \quad and \quad q = \frac{3\beta A_0^2}{4} \quad (9)$$

If $q = 0$ ($\beta = 0$), then $p = 1$ and $\omega_\Omega = \omega_0$, so that the harmonic oscillator is settled.

As a distinctive feature, the Mathieu resonator exhibits subharmonic solutions at half the driving frequency $\omega_\Omega = \omega_{1/2} = \omega_0/2\,(= \Omega/2)$ [13,14]. For the non-damped case ($\zeta = 0$), a very singular locus of resonant solutions occurs at $1 > q > 6$ (corresponding to relatively low nonlinearity; low β) [13]. This relevant region for Faraday wave piloting, or extraordinary subharmonic FW tongue ($\psi_{1/2}$ does appear here at $\omega_{1/2} = \omega_0/2$), actually coexists with a family of ordinary harmonic domains (the generating field ψ_0 appeared at $\omega_0, 2\omega_0, 3\omega_0, \ldots$). Although the presence of frictional damping can alter harmonic domains (causing broadening at $\zeta > 0$), however, the subharmonic feature prominently remains unaltered as an undamped resonance (parametric amplification for FWs) [18].

Very pertinently, the subharmonic wavefield $\psi_{1/2}$ elicits the DNLO system to resonate at half of the natural frequency, i.e. at $\omega_{1/2} = \omega_0/2$ (as observed in our NLSW experiments as an idiosyncratic FW distinctive; see Figure 2). In the DNLO-description, the wavefield generator depicts the subharmonic resonance as emerging from the intrinsic coupling between liquid inertia and the natural surface response (in the absence of external driving). The subharmonic field $\psi_{1/2}$ is coupled with the main wavefield ψ_0 via parametric modulation, see Eq. (6-7). The wavefield decomposition enlightens the link between the extrinsic harmonic resonance (the periodically forced DNLO field ψ_0), and the intrinsic sub-harmonic resonance due to field self-focusing (the Faraday wavefield $\psi_{1/2}$). Physically, the subharmonic resonance emerges from the periodic modulation of the Hookean spring response, which yields parametric resonance under frequency ($\omega_{1/2} = \Omega/2$) and amplitude conditions ($A_0^2\beta > 1$) [14-18]. The DNLO structure imposes a commons inertia-governed decay within the hybrid harmonic and sub-harmonic cascades governed by the effective mass m, independently of the specific natural response as embedded in the spring constant k (and the friction constant ζ) [15,16,19]. The appearance of these spectral features at specific FW-signature is analysed below.

1-6. Power spectral density

To describe the DNLO spectral responses in frequency domain, we follow the prevalent formalism considered in the surface wave turbulence (SWT) theory [3,9,10]. Likely, we compare theory and DNLO-predictions with measurements from NLSW experiments, which are harvested in terms of surface heights considered as wave amplitudes (more often used for analysis than the velocities directly measured by LDV). Here, we use the same definitions as introduced in the main text. This convenient representation deals with wavefield amplitude spectra as determined by the density of power per unit of frequency [3,9]:

$$PSD_{\psi}(\omega) \equiv \int \langle \psi(t)\psi(t+\tau) \rangle e^{-i\omega\tau} d\tau \sim \omega^{-\alpha} \tag{10}$$

This Fourier transform is called the power spectral density (PSD), which phenomenologically represents the hydrodynamic structure underlying the energetic cascade $PSD_{\psi} \sim \omega^{-\alpha}$ (as described by a Kolmogorov-like scaling law characterized by the decaying slope α, which describes the envelope of the spectral peaks). The PSD function measures the amount of covariance of the wavefield by each frequency ω, being determined by the height-to-height wavefield autocorrelation function calculated at periods spaced a lag time between events oscillating at frequencies $\omega = 2\pi/\tau$; for periodic wavefunctions:

$$\langle \psi(t)\psi(t+\tau) \rangle(\tau) \equiv \int_0^{\tau} \psi(t)\psi(t+\tau) dt \tag{11}$$

which deals with the time average $\langle \quad \rangle(\tau)$ calculated for each lag time τ.

In the experiments, we obtain the PSDs as the Fourier transform of the time series tracked for the surface roughness $\psi(\boldsymbol{r}, t)$, as observed at a given surface displacement $\boldsymbol{r}$ by the local LDV vibrometer; this is $PSD_{\psi}(\omega) = \mathcal{F}\{\langle \psi(\boldsymbol{r}, t)\psi(\boldsymbol{r}, t+\tau) \rangle\}(\omega)$ [20].

1-7. Model prediction: DNLO spectral slopes

We get DNLO-wavefield solution obtained upon numerical integration of Eq. (1) under synthetic AC forcing (as parametrized by the driving strength F_0 and frequency Ω). As far the reduced form of this equation is referred to unitary inertia ($m = 1$), all the other systemic parameters are varied by reference to unity. Only stationary solutions compatible with conserved energy initial conditions are considered, in agreement with the sustained character of the real-world realizations (unstable branches are discarded

since they do not represent physical situations considered in this work). To perform reliable comparisons with experiments, the PSDs are computed on the base of Eq. (10-11) for trajectory tracks $\psi(t)$ obtained from the above scheme as implemented in Wolfram Mathematica [21]. We numerically perform the integration by invoking the autocorrelation theorem based on the Parseval's identity [22]:

$$PSD_{\psi}(\omega) \equiv \int \langle \psi(t)\psi(t+\tau) \rangle(\tau) e^{-i\omega\tau} d\tau = \mathcal{F}\{|\psi(t)|^2\}(\omega) \quad (12)$$

where $\mathcal{F}\{f(t)\}(\omega)$ is calculated as the Fourier transform of the time series; this is:

$$PSD_{\psi}(\omega) = \int |\psi(t)|^2 e^{-i\omega t} dt \quad (13)$$

with the units of power (experimentally measured in $m^2\ Hz^{-1}$). All the computational codes are available upon request to the authors.

In order to compare calculated DNLO-spectra with the experiments, we have performed computational calculations covering the relevant parameter space for variable β and ζ (inertia fixed at $m = 1$). Supplementary Figure N2 shows typical results for parametric resonances with subharmonic response.

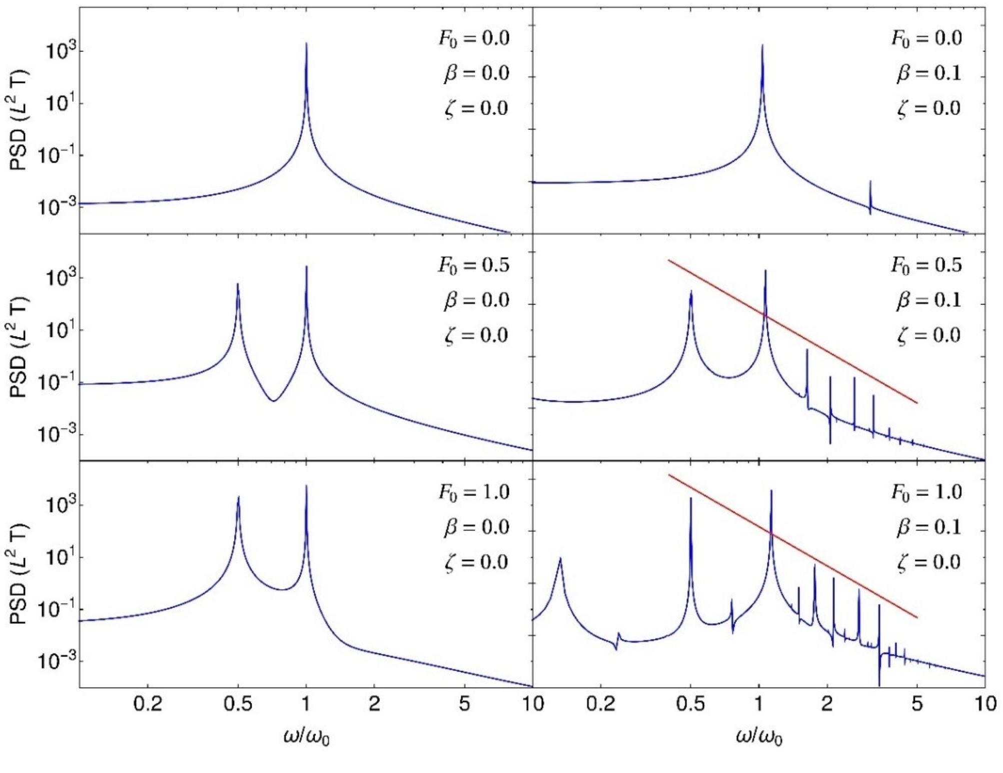

Supplementary Figure N2. Power spectral density for DNLO parametric resonance at increasing forcing ($\omega_0 = \Omega/2$ and $F_0 = 0 - 1$). Linear scenario ($\beta = 0$; left panels); nonlinear scenario ($\beta = 0.1$; right panels). A hybrid nonlinear cascade made of ordinary harmonics ω_n, and extraordinary subharmonics $\omega_{n/2}$ of the driving force ω_0, do appear with increasing the driving force. The characteristic $PSD \sim \omega^{-5}$ spectral decay typical of Faraday waves at parametric resonance is observed

upon stiffening nonlinearity at sufficiently high driving force (red lines with an apparent -5 slope in the double log PSD-frequency plot).

In the absence of friction ($\zeta = 0$), the two distinctive FW-signatures are predicted within DNLO-PSDs in Supplementary Figure N2: a) An emergence of subharmonic response at $\omega_{1/2} = \omega_0/2$ upon increasing F_0 (as imposed by inertia ($m = 1$), even in the absence of rigidization ($\beta = 0$); b) The appearance, upon nonlinearity, of composite cascades of ordinary harmonics $o \equiv \{\psi_0\}$, and extraordinary subharmonics $e \equiv \{\psi_{1/2}\}$, both decaying as $PSD_{DNLO}(\omega) \sim \omega^{-5}$, with an $\alpha = -5$ spectral slope imposed by inertia (at $\beta > 0$). At sufficiently high nonlinearity, the presence of lower subharmonics ($\omega_{1/4}$, $\omega_{1/8}$,…) is also detected upon increasing driving. The simulated spectra are in quantitative agreement with experimental observations (see Fig. 2 in the main text). From a completely analytic approach below, we get further insight on the inertial character of the spectral FW-signature ($\alpha = -5$).

N1-8. DNLO harmonic balance: inertial and frictional signatures

Analytic expressions can be predicted for the PSD's envelopes in the theoretical DNLO setting. We exploit the harmonic balance to perform homotopy analysis between the driving force $F(t) = F_0 e^{i\Omega t}$ and the expected solution $\psi(t)$. Assuming oscillatory solutions with the oscillatory form $\psi(t) = \psi_0 e^{i\omega t}$, from Eq. (1) we get:

$$[-\omega^2 + i\omega(\zeta/m) + \omega_0^2]\psi(1 + \beta\psi^2) = F(t)/m, \qquad (14)$$

and multiplying this equation by its complex conjugate, one obtains:

$$|\psi^2| \equiv \psi^*\psi = \frac{F_0^2}{m^2[\omega^2 - \omega_0^2(1+\beta\psi^2)]^2 + \zeta^2\omega^2} \qquad (15)$$

For $\beta = 0$, it reduces to the energy spectrum of the forced harmonic oscillator [8]:

$$\psi_0^2(\omega) = \frac{F_0^2}{m^2(\omega^2 - \omega_0^2)^2 + \zeta^2\omega^2} \qquad (16)$$

By expanding Eq. (15), for relatively small nonlinearity ($\psi \approx \psi_0 + o(\psi_0^2)$ at $\beta > 0$), we get the approximate formula:

$$\psi^2(\omega) \approx \psi_0^2\left[1 - 2\beta\frac{\omega_0^2(\omega^2 - \omega_0^2)}{(\omega^2 - \omega_0^2)^2 + (\zeta/m)^2\omega^2}\psi_0^2 + o(\psi_0^4)\right] \qquad (17)$$

i.e. although strengthened by the stiffening term ($\beta\psi_0^2$), the DNLO energy is essentially determined by the frequency dependence of the function ψ_0^2 (forced oscillator), which

measures which frequencies contain the signal's energy (in units L^2). This is somewhat different to the power spectral density (PSD) as defined by Eq. (13), which is the measure of signal energy content per frequency; the PSD can be looked upon as a frequency-domain plot of power per unit Hz vs. frequency (in units $L^2 \cdot T$). While the energy spectrum in Eq. (15) estimate the area under the signal plot, the PSD assigns units of power to each unit of frequency and thus, enhances periodicities. Consequently, the DNLO power spectral densities can be calculated from the energy spectrum in Eq. (17) as:

$$PSD_\psi(\omega) = \frac{\psi^2(\omega)}{\omega} \approx \frac{F_0^2\left(1-2\Xi(\omega)\beta\psi_0^2+\cdots\right)}{\omega\left[m^2\left(\omega^2-\omega_0^2\right)^2+\zeta^2\omega^2\right]} , \tag{18}$$

which represents the spectral envelope under stiffening perturbation modulated by a form factor centred at the natural frequency ω_0:

$$\Xi(\omega) = \frac{m^2\omega_0^2\left(\omega^2-\omega_0^2\right)}{m^2\left(\omega^2-\omega_0^2\right)^2+\zeta^2\omega^2} \tag{19}$$

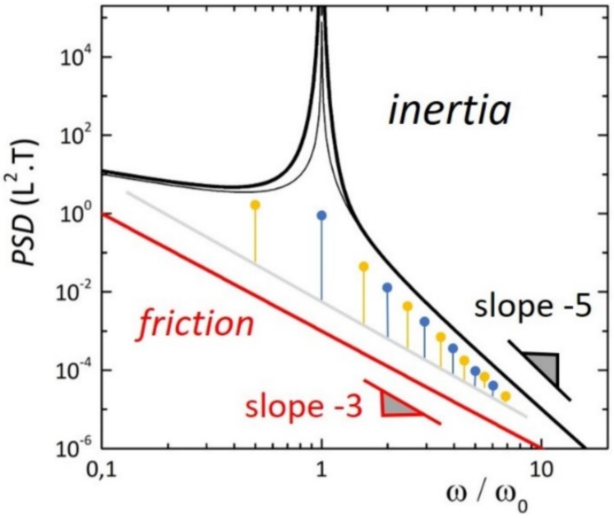

Supplementary Figure N3. DNLO spectral envelopes at harmonic balance. PSD profile for the Duffing oscillator at inertial resonance ($m = 1$) with a driving source at ω_0, Eq.(18) in black; linear resonance ($\beta = 0$; thin line); resonance focussing under Duffing stiffness, $\beta = 1$, under form factor Ξ as given by Eq. (19); thick line. A tail envelope with a characteristic -5 slope (FWs) is predicted for the discrete cascade of nonlinear harmonics: ordinary; $o \equiv \{\psi_0\}$ (yellow); extraordinary subharmonics; $e \equiv \{\psi_{1/2}\}$ (blue). In the non-inertial, pure frictional case ($m = 0$; $\zeta \neq 0$)) a -3 slope is predicted, in agreement with experiments at highly frictional conditions.

As shown by Supplementary Figure N3, two limiting behaviours are clearly discernible in the spectral tails of the PSD cascades:

a) Pure inertial ($m/\zeta \gg \omega_0$), with a PSD of amplitude proportional to the imposed acceleration ($\Gamma = F_0/mg$, in reduced units), decaying with frequency as:

$$PSD_\psi^{(inert)} \approx \frac{(F_0/m)^2}{\omega\left(\omega^2-\omega_0^2\right)^2} \sim \Gamma^2\omega^{-5} \tag{S20}$$

corresponding to the dominance of the extrinsic resonance between the driving source and the liquid inertia at the natural frequency ω_0 (with characteristic inertial signature $PSD_{\psi}^{(inert)} \sim \omega^{-5}$; see Supplementary Figure N3, black line).

As a formal connection with the well-known Phillips spectrum for random wavefields of crested waves generated by turbulent wind (under random driving), the $\alpha = -5$ spectral tail appears in Eq. (20), as a direct consequence of the detailed harmonic balance between the external driving force and the fluid inertia. In the equilibrium range of the spectrum, Phillips assumed a resonance mechanism at harmonic balance, i.e. when each component of the driving pressure moves as the component of the surface wavefield with the same frequency [23]. For random excitation on the order of the gravity force ($\Gamma \approx 1$), from dimensional analysis he obtained $PSD_{\psi}^{(Ph)} \approx \Gamma^2 \omega^{-5}$ [24], in agreement with the above calculation for monochromatic driving. Such a Phillips-like class of spectral tail has been detected as a distinctive for the FW regime not only in our experiments ($\alpha = -5$ at $\Gamma \geq \Gamma_F \approx 0.3g$), but also in previous studies with other FW-setups [25,26,27].

b) Pure frictional ($\zeta/m \gg \omega_0$), with a PSD of amplitude proportional to the driving velocity ($v = F_0/\zeta$), decaying with frequency as:

$$PSD_{\psi}^{(frict)} \approx \frac{(F_0/\zeta)^2}{\omega^3} \sim v^2 \omega^{-3} \tag{21}$$

corresponding to the dominance of internal viscous friction at balance with the driving force (with characteristic inertial signature $PSD_{\psi}^{(frict)} \sim \omega^{-3}$; see Supplementary Figure N3; red line). The characteristic $\alpha = -3$ spectral tail has been experimentally observed in the chaotic regime at $\Gamma \geq \Gamma_{chaos}$, where frictional stresses dominate (see Fig. 2 and Fig. 4 in the main text). A prediction is made from DNLO simulations at increasing friction, reported in Supplementary Figure N4.

1-9. Inertial FW domain: resonant mass-force spectrum

Supplementary Figure N4 shows inertial domain, FW-like simulations in the moderate stiffening regime ($\beta = 0.5$; where a resonance is dominated by bulk inertia). By finding nonlinear solutions locked at the natural frequency (at $\omega_0 \equiv \Omega$), we observe not only the piloting harmonic response (at $\omega_n = n\omega_0$) but also several subharmonics (at

$2\omega_0/3$, $\omega_0/2,\ldots$). By increasing the driving force, the system evolves from an incomplete, weakly nonlinear response constituted by the very first harmonics and subharmonics, to a well-developed nonlinear cascade with an inertial domain characterized by the slope-5 signature of FWs at resonance with bulk inertia (transparency window at no peak broadening; see lower panel).

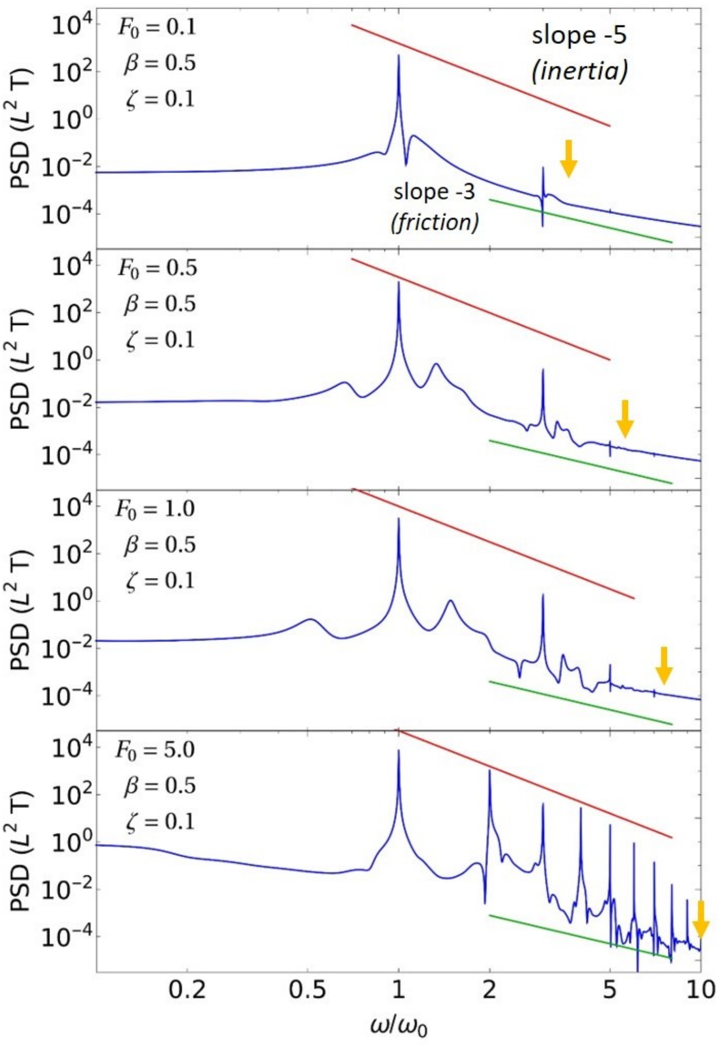

Supplementary Figure N4. Inertial domain of nonlinear FW cascades for the DNLO model at natural resonance with the driving force ($\omega_0 \equiv \Omega$). The PSDs are evaluated from nonlinear trajectories at increasing forcing ($F_0 = 0 - 5$). We consider the case of inertial response ($m = 1$) at moderate nonlinearity ($\beta = 0.5$) and low friction ($\zeta = 0.1$).The inertial domain, or transparency window, appears as a succession of very narrow resonances able to persist frictional death at higher frequencies. The spectral decay controlled by inertia is marked in red ($PSD{\sim}\omega^{-5}$); frictional control is marked in green ($PSD{\sim}\omega^{-3}$). An energy blue shift with increasing the pumped energy is observed in the upper frequency cut-off (see arrows). The frictional red shift can be observed under increasing friction (see Fig. SN5)

The role of viscous friction in killing the FWs at high frequencies is evident (Supplementary Figure N4; lower panel). Although the inertial cascades emerge composed by more and more intense harmonics with increasing F_0, a frictional death occurs at high frequencies (as observed in experiments; see Fig. 2 and Fig. 4 in the main text).

1-10. Loosing Faraday wavefield self-focussing: frictional death

The -5 spectral slope has been theoretically identified as a DNLO-wavefield attribute controlled by inertia (Phillips-like; at resonant balance with the driving source). This spectral behaviour is equivalent to the FW resonance observed in experiments (see Fig. 2 and Fig. 4 in the main text and Supplementary Figures 3-6). In addition, a transition to a -3 spectral slope has been realized as a progressive dominance of friction at loosing resonance. This behaviour is known in the DNLO's literature as a reverse bifurcation upon frictional defocusing [28].

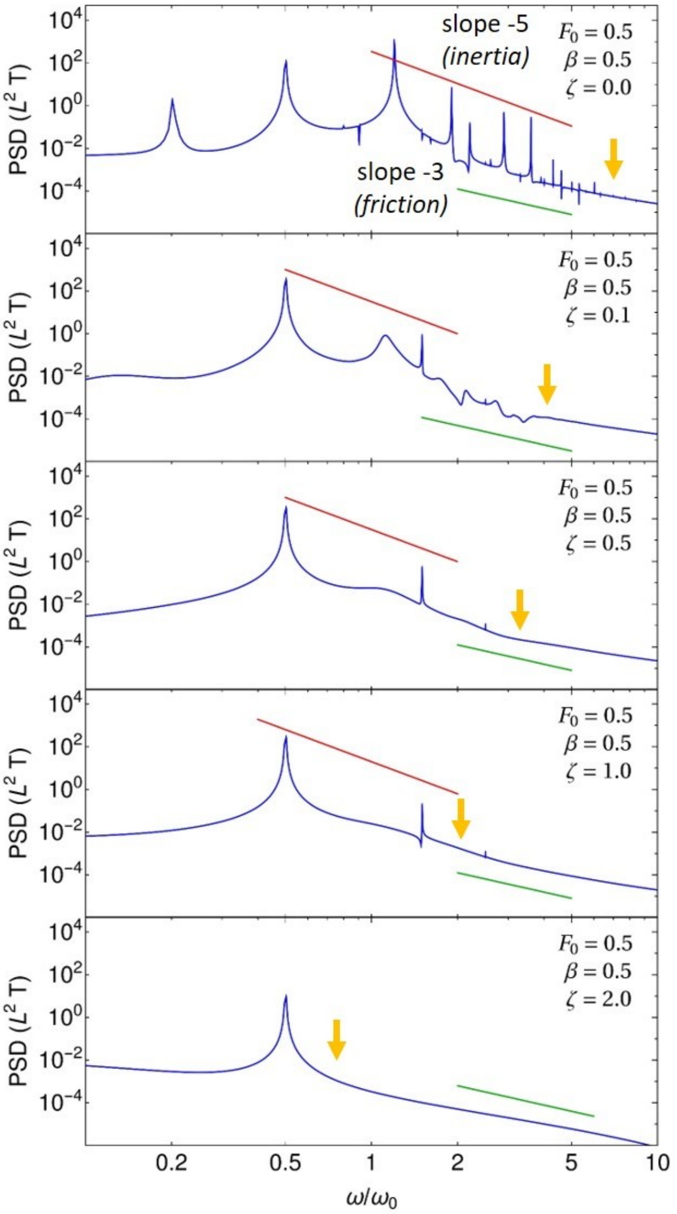

Supplementary Figure N5. Frictional death of nonlinear hybrid cascades for the DNLO model at parametric (subharmonic) resonance $\omega_0/2 = \Omega$. The PSDs are evaluated from nonlinear trajectories at increasing friction ($\zeta = 0 - 2$). We consider the case of inertial response $(m = 1)$ built up as an ordinary cascade of nonlinear harmonics appeared at moderate nonlinearity ($\beta = 0.5$), superposed to an extraordinary (subharmonic) cascade appeared upon parametric resonance at relatively low driving force ($F_0 = 0.5$). The inertial resonance characterized by the -5 spectral slope (red line) is progressively substituted by a -3 tail (green line), which imposes a spectral signature for the frictional death of the two cascades. Both features can coexist in a range since the piloting field is strongly damped by viscous friction whereas the subharmonic field does persist amplified under parametric resonance (as experimentally observed; see Figs. 2 and 4). An evident red shift of the inertial window occurs with increasing friction (see arrows for the cut-off frequency).

The Supplementary Figure N5 shows representative DNLO-results for inertially resonant FWs at frictional degeneracy. Upon increasing friction ($\zeta = 0 - 2$), we

observe dissipation spreading over the composite wavefield made of ordinary harmonics $o \equiv \{\psi_n\}$ at frequencies $\omega_n = n\omega_0$, and extraordinary subharmonic states $e \equiv \{\psi_{n/2}\}$ at $\omega_{n/2} = n\omega_{1/2}$. Whereas the harmonic modes progressively damp (at peak broadening proportional to ζ), the subharmonic modes keep undamped at resonance (regardless of ζ). Under these circumstances, the DNLO wavefield undergoes progressive frictional losses within the ordinary component (ψ_0); however, the subsidiary subharmonic component does persist ($\psi_{1/2} > \psi_0$). Although the relative amplitudes of both wavefields vanishes upon friction, the o-cascade rapidly overdamps into a ω^{-3}-spectral decay but the e-cascade persists undamped with the ω^{-5}-feature remaining (FW-like). These simulations evidence the superposition of the two different subfields in Eq. (2) into hybrid FW-cascades as observed in experiments; see Fig. 2 and Fig. 4 in the main text. On the one hand, the generating wavefield ψ_0 behaves as a forced NLO that plays a skeletonizing role as a waving background that evolves steadily although incoherently at low forcing, see Eq. (5). On the other hand, $\psi_{1/2}$ endows the parametric resonance piloting the subharmonic response that supports a coherent FW-structure at increasing nonlinearity, see Eq. (6). The DNLO structure supports these two fields superposed (as shown in Supplementary Figure N5). Keeping the DNLO parameters fixed except for effective friction, we have determined the competition between self-focusing and dissipation features in a transitional domain from steady waving to unsteadiness (as observed in NLSW experiments at the onset of the chaotic regime; see Figs. 2 and 4). A DNLO-guided chaotic mechanism for FW-death under frictional ψ_0-defocusing is thus suggested at increasing velocity. This progressive loss of FW-field coherence was observed in the chaotic states at high driving force (see Fig. 2 and Fig. 4; bottom panels, and discussion below on Landau's chaos). Such deterministic class of DNLO-guided transition to chaos should be, however, different to the incoherent class of wave turbulence argued to underly the disordered states observed below the FW-threshold (see upper panels in Fig. 2 and Fig. 4; the KZ-cascades of NLSWs decaying at $PSD \sim \omega^{-4}$ for GWs, or at $PSD \sim \omega^{-17/6}$ for CWs; see also Supplementary Figure 3. That KZ-regime will be discussed in Supplementary Note 2 as a special case of non-inertial (incoherent) DNLO dynamics governed upon the nonlinear Schrödinger (NLS) equation [3].

1-11. Transition to unsteady chaotic regime: Landau's spectrum

Our experiments showed a transition from a steady FW-regime of discretized modes ($\Gamma_F \leq \Gamma \leq \Gamma_{chaos}$), to an unsteady chaotic regime of unstable NLSW motion above a force threshold at $\Gamma_{chaos} \approx 1$. Whereas the hybrid cascades of harmonics $\{\psi_n\}$ and subharmonics $\{\psi_{n/2}\}$ coexist superposed at isolated frequencies in the FW-regime, at $\Gamma \geq \Gamma_{chaos}$ we observe a continuous spectrum that contains many modes filling the spectral density of states (see Fig. 2 and Fig. 4; lower panels). As envisaged by L.D. Landau on his Paper #52 On the Problem of Turbulence [29,30], this class of continuum spectrum does appear as a signature for the deterministic initiation of a progressive unsteadiness of the principal motion composed of discrete modes at isolated frequencies [29].

Building upon the steady motion that forms the discrete spectrum for the principal stationary motion, Landau supposed that the frequencies of the continuum spectrum correspond to unstable modes created as new degrees of freedom from the former discrete modes, i.e. the turbulent flow field can be expressed as a Fourier series $\psi = \sum_p \psi_p$, with time-varying components of the form $\psi_p \sim e^{i\omega t} e^{\gamma t}$ (γ is a rate of unsteadiness). As turbulence increases, the number p of degrees of freedom becomes likewise progressively large, thus leading chaotic turbulence once superposed [29]. As a matter of fact, the modulus ψ^2 of the nonstationary chaotic motion should not increase infinitely, but rather remains bounded to a certain upper limit for stability, i.e. $\psi^2 \leq \psi^2_{max} \approx \psi^2_{chaos}$ (corresponding to $\Gamma \approx \Gamma_{chaos}$). Nearly above the critical threshold ($\Gamma \geq \Gamma_{chaos}$), this perturbed bound is yet small and close to the principal solution for the stationary flow, i.e. $\psi_{chaos} \approx \psi + \delta\psi$. Said otherwise, the transitional pathway from steadiness to chaos should be deterministic in nature, thus described by a kinetic equation that Landau assumed with a harmonic structure at even expansion term not vanishing by time averaging; specifically, he proposed [29]:

$$\frac{d|\psi|^2}{dt} = 2\gamma|\psi|^2 - \lambda|\psi|^4 \qquad (22a)$$

where the kinetic constant γ determines a rate for the loss of steadiness. The positive constant λ plays a softening role as a negative stiffness in the DNLO ($\beta < 0$), i.e. the quartic term in Eq. (22a) contributes field steadiness under wave focussing. Since $d|\psi|^2/dt = 2\psi\dot{\psi}$, Eq. (22a) takes the same form as a DNLO devoid of inertia at

effectively negative spring rigidity ($\gamma = -k/\zeta$) and negative stiffness (softness $\lambda = -\beta$); this is:

$$\frac{d\psi}{dt} - \gamma\psi(1 - \lambda|\psi|^2) = F(t)/\zeta \quad (22b)$$

The DNLO equivalence of the Landau's chaotic unsteadiness can be interpreted as a repulsor-like dynamic evolution of an inverted oscillator. The strength of the repulsive barrier is determined by the negative ratio of the spring constant to friction; the higher $-k$, and/or the lower ζ, the larger the observed unsteadiness (increasing γ). The Eq. (22b) shows increasing viscosity to cause progressive steadiness (by decreasing effectively the driving force). Softening nonlinearity (Duffing-like; $\beta < 0$) takes here the role as an unsteadiness source reinforcing the repulsive barrier to sharpen ($\lambda > 0$). Because the derivative in Eq. (22a) is carried out over such time intervals as are small in comparison with γ^{-1}, one founds [29]:

$$\frac{1}{|\psi|^2} = \frac{\lambda}{2\gamma} + Ae^{-2\gamma t} \quad (23)$$

where $|\psi|^2$ increases asymptotically up to an upper limit $|\psi|^2_{max} = 2\gamma/\lambda$.
Landau argued about a critical threshold Γ_{chaos}, for the larger the amount of unsteadiness, i.e. $\gamma \sim (\Gamma - \Gamma_{chaos})$. The exponential growth for the loss of steadiness stablishes the distinctive spectral signature for the catastrophic transit from steady to chaotic flow; by taking the Fourier transform for the time-dependent part in Eq. (23), the Landau's PSD is obtained with a Lorentzian shape:

$$PSD_{\psi}^{(chaos)} = \frac{2\gamma/\lambda}{4\gamma^2 + \omega^2} \sim \lambda^{-1}\omega^{-2} \quad (24)$$

with zero-frequency amplitude decreasing with stiffness (as $\lambda^{-1} \sim \beta^{-1}$), and a characteristic ω^{-2}-tail decaying along a frequency bandwidth of length 2γ, given by the unsteadiness rate. This Landau's chaotic feature has been realized in our experiments at driving force above a threshold for chaos ($\Gamma > \Gamma_{chaos}$; Figs. 2 and 4).

1-12. DNLO chaotic regime: Landau's mode superposition

We have checked the above Landau's turbulence in the DNLO setting. Figure SN6 shows a series of simulations at subharmonic resonance. A transitional scenario is evidenced with increasing external driving force even at low nonlinearity ($\beta \approx 0.01$); we detect two characteristic features: a) New and more offspring modes observed to

emerge as satellites around the discrete modes that constitute the principal response (Supplementary Figure N6 a); b) Mode-superposition in a Lorentzian continuum with the ω^{-2}-decay being the more apparent the higher the unsteadiness (Supplementary Figure N6 b).

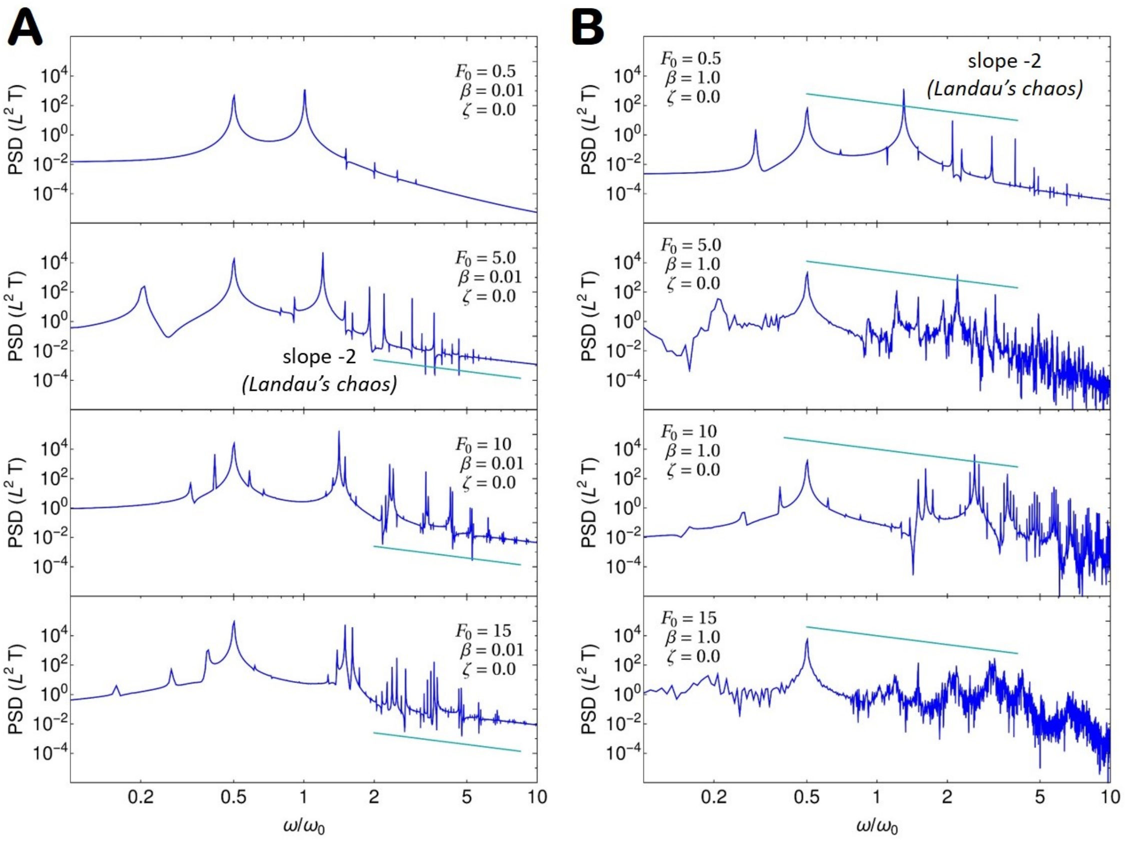

Supplementary Figure N6. DNLO realization for the Landau's scenario for chaotic turbulence at vanishing friction. We probed a simulation setting for a fully inertial DNLO ($m = 1$), frictionless ($\zeta = 0$) at parametric resonance ($\omega_0/2 = \Omega$). With increasing driving force ($F_0 = 0.5 - 15$): A) Low stiffening nonlinearity (small unsteadiness; $\beta = 0.01$); B) High stiffening nonlinearity (big unsteadiness; $\beta = 1$). The characteristic -2 Landau's slope typical for unsteady turbulence is detected with increasing unsteady mode superposition (cyan line).

The sensitivity of these chaotic regimes to friction is analysed in Supplementary Figure N7, which plots hybrid DNLO cascades simulated with increasing forcing at parametric resonance; either for relatively low friction ($\zeta = 0.01$), or in the high viscosity regime ($\zeta = 2$).

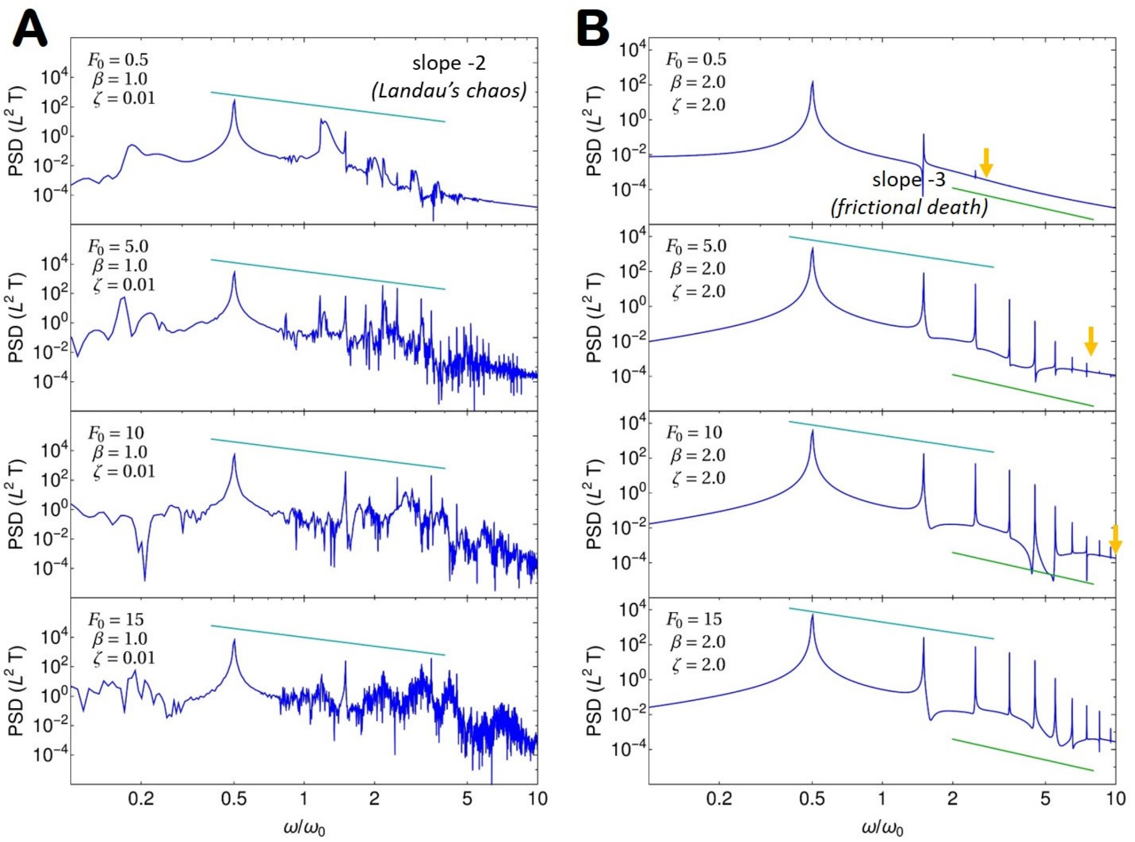

Supplementary Figure N7. Frictional sensitivity for Landau chaotic turbulence in a DNLO realization at FW-like parametric resonance. We probed variable nonlinearity and friction in a simulation setting for a fully inertial DNLO ($m = 1$) at subharmonic resonance ($\omega_0/2 = \Omega$). With increasing driving force ($F_0 = 0.5 - 15$): A) Moderate stiffening nonlinearity ($\beta = 1$), and very low friction ($\zeta = 0.01$). The characteristic -2 Landau's slope is detected with increasing mode superposition at increasing driving (cyan line). B) High stiffening nonlinearity ($\beta = 2$), and high friction ($\zeta = 2$). The dominant -3 frictional feature at high frequencies (green line) starts to coexist with the characteristic -2 Landau's slope appeared upon increasing mode superposition at lower frequencies (cyan line). The higher the energy injected, the higher the cut-off frequency of the nonlinear cascades (yellow arrows).

The model predicts a friction-regulated mode superposition within the lower harmonics and subharmonics, which induces a chaotic response characterized by lower spectral slopes nearly decaying as $PSD_{chaos} \sim \omega^{-2}$ (much slower than the canonical subharmonic FW resonance governed by inertia; this is $PSD_{FW} \sim \omega^{-5}$). However, friction causes mode killing even at very low viscosity (see Supplementary Figure N7 A), a distortion specially overwhelming for the weaker internal resonances between highly dissipative harmonics (see Supplementary Figure N7 B). Under high friction only the subharmonic skeleton is able to persist with a structure resembling the Landau spectrum ($PSD \sim \omega^{-2}$ at $\omega < \omega_F$), following a terminal regime till reaching frictional death ($PSD \sim \omega^{-3}$ at $\omega > \omega_F$). These DNLO-features agree with the spectral features observed in experiments at the onset of the chaotic regime.

As a final remark, the leading role of subharmonic resonances imposing an unsteady structure quite immune to friction has been evidenced in the DNLO. Unsteadiness appears as the natural consequence of nonlinearity making new unstable modes to emerge in a turbulent continuum. In the same way to the Landau's mechanism for the appearance of turbulence, nonlinear stiffening intervenes for increasing driving force as more and more new periods appear in succession. Viscous drag plays a key role at imposing a frictional death for this determinism. This complexity is encoded in the DNLO and can explain the transition from the coherent Faraday regime to the chaotic state observed in experiments.

Supplementary Note 2

Nonlinear water surface wavefields: DNLO unification.

2-1. Discrete KZ-cascades in the massless DNLO wavefield: One-dimensional MMT turbulence.
2-2. Massless MMT-DNLO at strong turbulence: self-focused Schrödinger regime.
2-3. Mass and stiffness are at the core of the ordering wave interaction: a reliable FW evolver for hydrodynamic crystal condensation.
2-4. NLSW unification into the DNLO model: dynamic NLSW states.

The DNLO simulations in Supplementary Note 1 convey on the reliability of bulk inertia and surface stiffening features as a minimal description for NLSW wavefields with a self-focusing dynamics leading discrete-time resonances that can result in hydrodynamic skeletonization. Although these minimal organizational principles tell us on the several energy distribution pathways among the possible states discretized in time by resonance, however, nothing is said about the possible spatial structure imposed by wave dispersion.

Experiments with discrete NLSWs have evidenced a superposed level of spatial organization revealed to play a key role in determining their spatiotemporal structure; from the random wavefields endowed with a KZ-scaling dependent of wave dispersion (gravity-like $\sim \omega^{-4}$, or capillary-like $\sim \omega^{-17/6}$), up to the organized FW fields with a highly resonant time structure (decaying as $\sim \omega^{-5}$), but a spatial edifice determined by

the particular regime of surface wave dispersion (three-folded triangular symmetry for GW's and four-folded square symmetry for CWs). Any theory should, therefore, take comprehensive account of dispersive wave interactions leading to spatial organization compatible with the dynamical principles contained in the DNLO model. A unified wisdom including the sub-limiting case of random KZ-wavefields would be, of course, strongly acknowledged. Before taking steps towards such a unification, we will first discuss the weakly turbulent (incoherent) wavefields as a special DNLO's class at neglected mass-force resonance; as a preliminary, KZ-turbulence is argued with a massless DNLO as described by Zakharov and Filonenko in view of the nonlinear Schrödinger (NLS) equation [10].

2-1. Discrete KZ-cascades in the massless DNLO wavefield: One-dimensional MMT turbulence.

Weak NLSW turbulence is known to take place in systems of nonlinear dispersive waves [3,9,10]. The energy transfer between waves compatible with their dispersion structure occurs mostly among resonant sets of waves in a random mixing of phases. Zakharov showed the weak turbulent dynamics to satisfy linear evolution equations for the mode amplitudes; building upon the Hamilton equations through of a perturbed interaction $\mathcal{H} = \mathcal{H}_2 + \mathcal{H}_{int}$ at their kernel, the dynamic equation primarily describes kinetic driving upon wave action $\mathcal{H}_2 = \sum_k \omega_k a_k^2$, plus mode-mode interactions $\mathcal{H}_{int}$; in Fourier space [3,9,10]:

$$i\frac{\partial a_k}{\partial t} = \omega_k a_k + \mathcal{Z} - \mathcal{D}_k + \mathcal{F}_k, \qquad (24)$$

For weak and random wave interaction, the conservative interaction term is calculated under the Zakharov's integral, $\mathcal{Z} = \int \mathcal{H}_{int}(\boldsymbol{k}_1, \boldsymbol{k}_2, \ldots) d\boldsymbol{k}_1 d\boldsymbol{k}_2 \ldots$ [3,10]. The kinetic equation depicted in Eq. 24 also includes a sink for frictional damping $\mathcal{D}_k$, in addition to the external driving source $\mathcal{F}_k$. The inviscid KZ-cascade captures an average flux of energy within the respective integral of motion [31], which is calculated for a continuum of coupled modes across the inertial interval [3,10]. The dynamic status of such a system is called turbulent as far the large wavelength greatly differs from the smaller dissipation scale (as occurred in turbulent flows [32]). The cascade's concept fixes the macroscopic manifestation of turbulence: the mean rate of the energy pumping does not depend on viscosity along the inertial domain of turbulence [32].

Assuming weak interaction at lowest order perturbative expansion ($\mathcal{H}_{int} = \mathcal{H}_3 + \mathcal{H}_4 + \cdots$), Zakharov described a transport of the integral of motion propagating wave action and wave collision interactions from the driving source to small scales. For the cascade picture to be valid, the collision integral assumes locality in k-space [3]. Under random forcing ($\mathcal{F}_k$ chosen as a Gaussian distribution of noise), at the inertial interval (transparency window at no dissipation $\mathcal{D}_k = 0$), for leading interaction terms with a spatial structure compatible with the wave dispersion symmetry, the derived KZ-spectra render into the well-known classes of Kolmogorov-Zakharov (KZ) scaling: a) $PSD_{CW} \sim \omega^{-17/6}$ for three-wave interaction of CWs dispersing as $\omega \sim k^{3/2}$, and b) $PSD_{GW} \sim \omega^{-4}$, as corresponds to the four-wave collisional dismutation for GWs dispersing as $\omega \sim k^{1/2}$ [3,9]. These KZ-genuine regimes have been detected under monochromatic excitation in a forcing window below the Faraday threshold (see Figures 2 and 4; upper panels). For noninteracting waves, however, only the quadratic wave action might be accounted for, i.e. $\delta\mathcal{H}/\delta a_k = \omega_k a_k$ ($\mathcal{H}_{int} = 0$) [3,9]. In this case the equation of motion takes the same form as a forced, damped oscillator devoid of bulk inertia. In terms of field variables, the noninteracting version of Eq. (24) becomes:

$$\zeta \frac{\partial \psi_k}{\partial t} + \omega_k^2 \psi_k = F_k, \qquad (25)$$

with a friction coefficient ζ that recapitulates the damping term $\mathcal{D}_k$.

Since we are here interested in a unifying framework, we refocus the simplified description based on NLO-equations at one dimension. A family of dispersive equations of the NLO-class was developed by Majda, McLaughlin and Tabak, namely the MMT model [33]. They assessed the validity of the statistical theory of weak turbulence for one-dimensional waves in the KZ-regime at random forcing; the generalized MMT equation is structured as [33,34,35]:

$$i\frac{\partial \psi}{\partial t} + \left(\frac{\partial}{\partial x}\right)^{\mu} \psi + \beta \left(\frac{\partial}{\partial x}\right)^{\nu/4} \left\{ \left| \left(\frac{\partial}{\partial x}\right)^{\nu/4} \psi \right|^2 \left(\frac{\partial}{\partial x}\right)^{\nu/4} \psi \right\} = F(t), \qquad (26)$$

where the exponent μ describes the spatial structure of the dispersive NLO. The non-dispersive case is $\mu = 0$, corresponding to constant propagation frequency (no spatial structure, e.g. the harmonic oscillator). The parameter β describes stiffening nonlinearity with a spatial structure embedded in the exponent ν; the relative $\mu = 0$

and $\nu = 0$ corresponds to the DNLO without inertia, $m = 0$ in Eq. (1). Interestingly, $\mu = 2$ describes Fickian-like dynamics.

An essential behavioral difference arose between MMT and KZ turbulence; the PSD-scaling predictions differed very significantly with large scale forcing and dissipation [33-35]. A source of failure of KZ-conditions in MMT-NLOs was identified as a lack for the locality assumption for wave interactions inside resonant sets [35]; indeed, basic MMT does not consider intrinsic couplings explicitly.

2-2. Massless MMT-DNLO at strong turbulence: self-focused Schrödinger regime

When nonlinearity becomes dominant, or exceeds dispersion, different organized structures are predicted to occur because of strong turbulence (even at no bulk inertia; $m = 0$) [4,9]. They belong, however, to the MMT class ($\mu = 2$ and $\nu = 0$) of nonlinear wave packets described by the forced NLS equation [10]:

$$i\frac{\partial\psi}{\partial t} + \nabla^2\psi + \beta|\psi|^2\psi = 0 \tag{27}$$

in which the nonlinear term accounts for wave attraction (wavefield self-focussing under stiffening at $\beta > 0$). Wave repulsion is described under softening (at $\beta < 0$). Weak wave turbulence resumes at $\beta = 0$. An evident connection exists between the DNLO oscillator in Eq. (1), the MMT model for wave turbulence in Eq. (26), and the NLS equation in Eq. (27). The MMT diffusive class ($\mu = 2$ and $\nu = 0$) reduces to NLS equation with forcing:

$$i\frac{\partial\psi}{\partial t} - iD\frac{\partial^2\psi}{\partial x^2} + \beta|\psi|^2\psi = F(t), \tag{28}$$

which conserves the same structure as a DNLO without inertia ($m = 0$), with a diffusion coefficient determined by the ratio of elastic response to friction ($D \sim k/\zeta$). We have further elaborated on the NLS structure. Supplementary Figure N7 shows DNLO simulations at no inertia ($m = 0$), which highlight this transit (equivalent to spatial homogeneous NLS equation; $D(\partial^2\psi/\partial x^2) = k\psi$ in Eq. (28).

The emergence of wide NLS cascades is evident at increasing driving (Supplementary Figure N8 A). As expected, a frictional control is observed at fully developed nonlinearity compatible with the ω^{-3}-envelope, see Eq. (21). Increasing friction causes

the cascades to die at progressively lower frequencies (see Supplementary Figure N8 B). We observe a transition between a well-developed cascade at low friction to a pyramidal “wedding cake” organization around the leading mode at high friction (frozen turbulence) [36]. These simulations devoid of inertia evidence differences with respect to the DNLO inertial case studied in Supplementary Note 1 (showing the FW-distinctive ω^{-5}-envelope imposed by inertia; see Supplementary Figure N3).

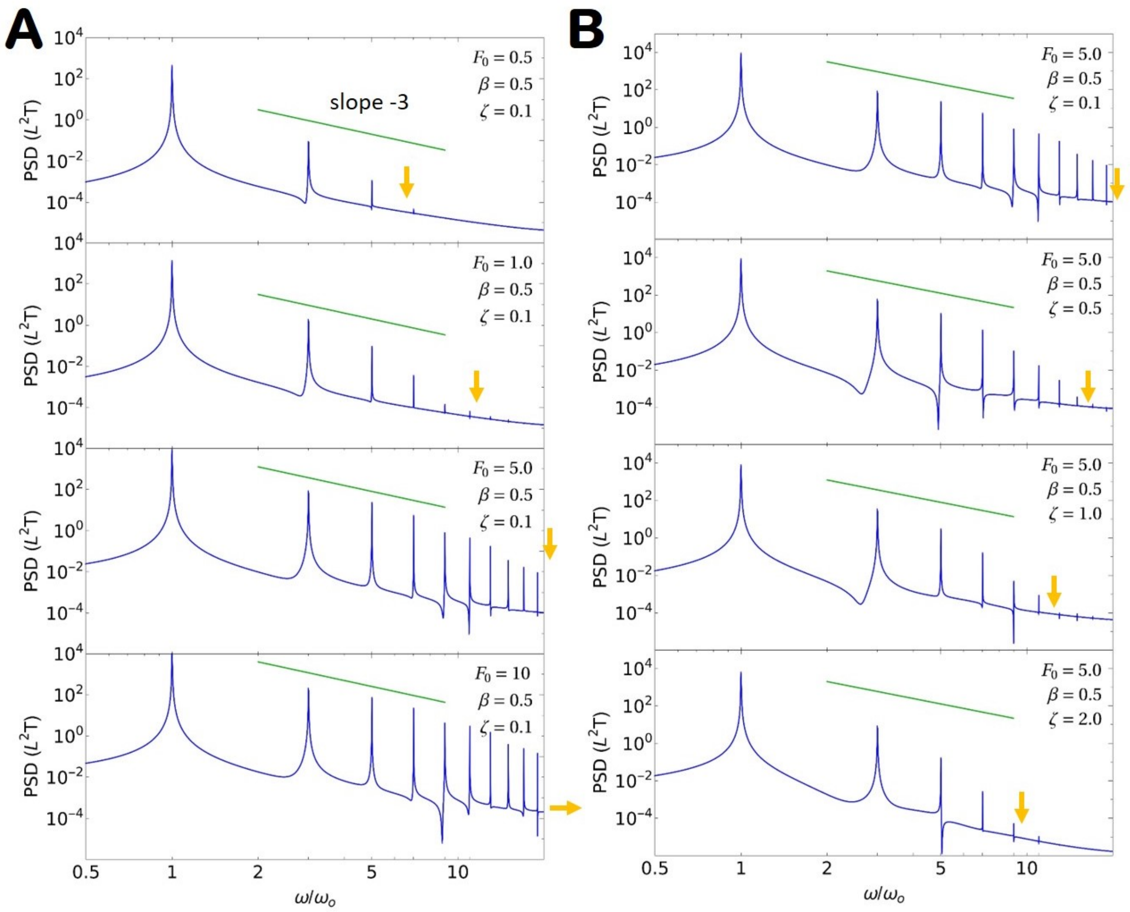

Supplementary Figure N8. Nonlinear Schrödinger scenario for strong wave turbulence. We probed NLS dynamics in the DNLO setting for no inertia ($m = 0$) at harmonic resonance ($\omega_0 = \Omega$). A) Emergence of turbulent NLS cascades with increasing driving force ($F_0 = 0.5 - 10$). A frictional dominated scaling is reached at high driving (slope -3: green line). B) Influence of increasing friction over strong NLS turbulence (slope -3: green line). A progressive frictional death occurs upon increasing $\zeta (= 0.1 - 2)$.

2-3. Mass and stiffness are at the core of the ordering wave interaction: a reliable FW evolver for hydrodynamic crystal condensation

The forced NLS equation describing KZ-developed turbulence recapitulates into a DNLO devoid of inertia. From the evidence raised, one can hypothesize that typical turbulent states (phase incoherent) correspond to a balance between dispersion and

localized nonlinearity in k-space [3-9]. Induced wave ordering (phase coherence) has been evidenced as a consequence of the detailed resonant balance between external forcing and inertia. The DNLO momentum term has been revealed as the crucial ingredient for predicting the ω^{-5}-feature typical of FW-spectra at parametric resonance under external forcing; lack of inertia causes, however, the extrinsic resonance quickly to dissipate into an overdamped ω^{-3}-regime (see Supplementary Figure N3). Looking at Eq. (1), one identifies the strong condition $m\ddot{\psi} > F$ as a threshold for inertial resonance; i.e. FW formation occurs only above the critical amplitude $\psi > \psi_F = F_0/m\omega_0^2$, or at $\Gamma > \Gamma_F = \psi_F\omega_0^2/g = F_0/mg$, in terms of the driving acceleration. Our experiments have revealed the condition $\Gamma_F \approx 0.1$, which implies a driving force higher than 10% the mobilized liquid weight involved at FW resonance ($F_0 > 0.1mg$). The upper FW-border was detected at $\Gamma = \Gamma_{chaos} \approx 1$, which corresponds to $F_{chaos} \approx mg$, i.e. FWs cannot longer exist provided inertia is overcome by the driving force. Under these parametrically resonant conditions ($\Gamma_F \leq \Gamma \leq \Gamma_{chaos}$), all the excited FWs resonate at the natural frequency fixed by the driving force, and at its higher harmonics and subharmonics (at wavelengths that are integer divisors of a fundamental wavelength $\lambda_{1/2}$ determined by the piloting wave of frequency $\omega_{1/2}$), oscillating out-of-phase with respect to the driving frequency ω_0. They become, consequently, very prone to develop spatial organization of wavelength inversely proportional to their frequencies. At high levels of non-linearity, the different signs of β could describe different physics:

a) Stable FW-condensates under surface stiffness (at $\beta > 0$), such as the hydrodynamic crystals made of coherently bounded waves discovered in this work.

b) Surface instability at wavefield defocusing ($\beta < 0$); under this hypothetical condition, e.g. in modulated surface wave separation [37], wave packet collapse into single states becomes possible.

At insufficient self-focusing below a threshold for wavefield condensation ($\beta \leq \beta_{cond}$), FWs should be unable to spatially organize as observed in experiments with weaker surface stiffening agents that aescin (see Fig. 5c). With the strong surface stiffener aescin, however, hydrodynamic crystals do appear at sufficient rigidization ($\beta \geq \beta_{cond}$). This condensation is the natural consequence of self-focused wavefield

conditions causing coherence to spatially organize the wave pattern with a permanent form, which is compatible with the dispersion structure of the piloting wave.

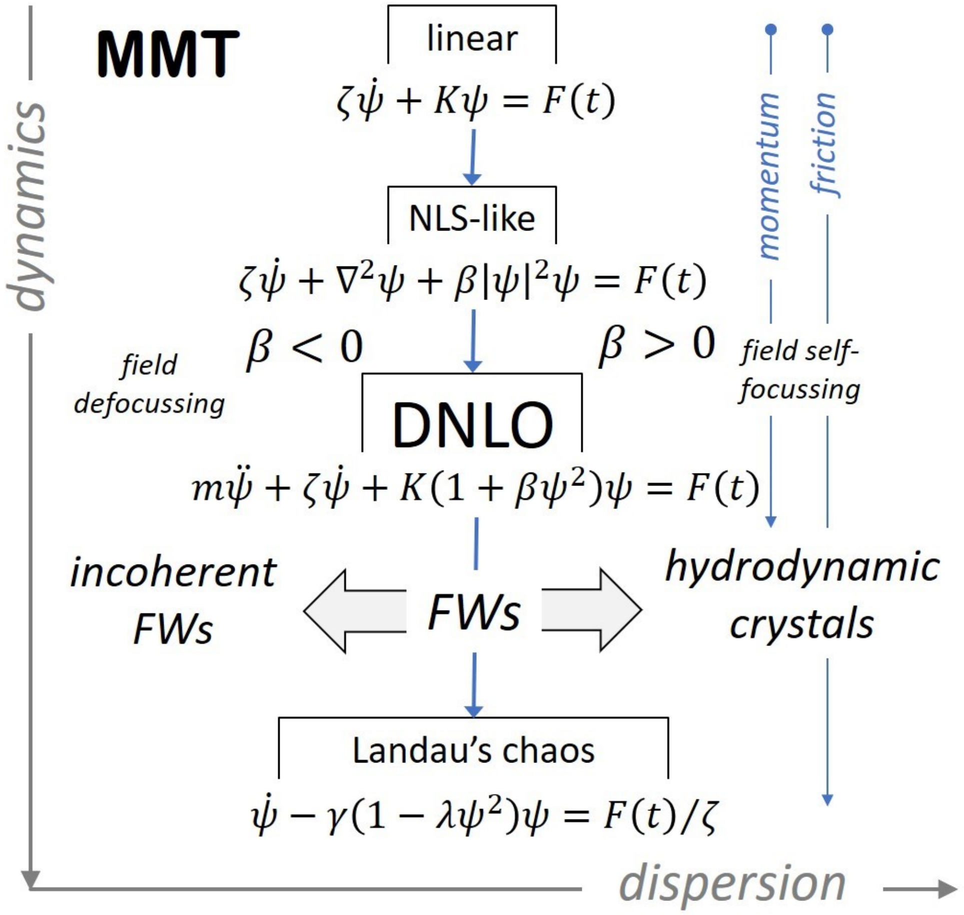

Supplementary Figure N9. Unification schematics for DNLO-NLSWs in the generalized MMT framework. The fundamental dynamic DNLO skeletonization is determined as a trade-off between the external driving force $F(t)$, systematic momentum (as determined by the inertial mass m), and frictional stress (fixed by viscous drag ζ). A transverse timeline of NLSW state evolution is envisaged as a DNLO dynamics at modulated inertial response and non-linearity; from top to bottom: 1) Linear response ($m = 0$; $K > 0$; $\beta = 0$). 2) NLS regime from weak to strong turbulence ($m = 0$; $\beta \neq 0$). 3) Forced inertial DNLO for parametric resonance leading FW formation ($m = 1$): whereas surface stiffening causes wavefield self-focusing (the necessary condition for hydrodynamic crystal formation; $\beta > 0$), surface softening elicits phase decoherence ($\beta < 0$). 4) Further forcing causes the system to enter the Landau's regime of chaotic turbulence, which resumes into a non-inertial inverted DNLO ($m = 0$ and $k < 0$) at unsteady chaotic evolution in a repulsive barrier regulated by friction; the unsteadiness rate is determined by the inverted oscillator constant referred to the friction coefficient ($\gamma = -k/\zeta$); surface Duffing stiffening refocuses the wavefield thus relaxing chaotic unsteadiness ($\lambda = -\beta$). The spatial structure of the NLSW states is envisaged as a transversal infrastructure along the dynamic DNLO skeleton. Wave dispersion appears at the core of this superposed spatial skeletonization as encoded in the MMT exponents for the spatial derivatives (μ for the linear term and ν for the nonlinear wavefield self-interaction; see Eq. S26). The genuine non-dispersive DNLO is identified as the simplest MMT class ($\mu = \nu = 0$). As a well identified spatially organized status, the NLS states of strong turbulence are stablished as a diffusive structure ($\mu = 2$; $\nu = 0$). Other classes of spatial organization are available among the family of MMT relatives.

2-4. NLSW unification into the DNLO model: dynamic NLSW states

In a step forward the generalized wisdom of the physics underlying the discrete NLSW cascades here involved, the discretized wavefield in Eq. (1) should fulfil the Navier-Stokes equations subjected to appropriate boundary conditions. These are primarily given by the extrinsic spatiotemporal structure of the driving force $F(\boldsymbol{r}, t)$. In our NLSW setting this force is oscillatory, spatially homogenous and perfectly monochromatic (as an intensive AC source $F(\boldsymbol{r}, t) = F_0 cos(\Omega t)\ \forall \boldsymbol{r}$). The essential component for ordering discretization is bulk inertia, which is recapitulated in the DNLO under the momentum term as parametrized by the apparent inertial mass (m). Determinant too are the constitutive relationships intrinsically connecting the wavefield structure with the underlying material properties [3,10] (here coarse grained under the DNLO parameters k and β). For the sake of clarity, we restrict here to a qualitative unification around the equivalent DNLO model. These ideas could pave the way towards a more quantitative theory of discrete NLSW fields covering the possible mechanical states available from the variable trade-off between external forcing, bulk resonating inertia and coupled nonlinear surface response. Such a unification has been depicted in Supplementary Figure N9 on the framework of MMT analytics.

Supplementary Note 3

Towards a classical field theory of hydrodynamic crystals: Faraday matter waves

3-1. NLSW state diagram upon discrete excitation: recapitulating bulk inertia and surface elasticity as FW fundamentals
3-2. Hydrodynamic crystalline state: FW-freezing under surface stiffening
3-3. DNLO-NLS equivalent scalar wavefield: wave equation
3-4. Wavefield discretization: elemental quantum of action; Klein-Gordon wave equation.
3-5. Linear Lagrangian: effective mass interaction
3-6. Accounting for DNLO-stiffening: quartic self-interaction
3-7. Faraday matter waves
3-8. Self-focused wavefield spatial structure: Lifshitz's field for Faraday waves

3-1. NLSW state diagram upon discrete excitation: recapitulating bulk inertia and surface elasticity as FW fundamentals

Here we recapitulate the connections between the DNLO predictions and NLSW states observed upon nonlinear resonance in our experiments at monochromatic driving. As an essential piece of understanding on why the hydrodynamic crystals are formed, Supplementary Figure N10 shows in phase diagrams how disorganized KZ-NLSW states can be frozen into ordered, FW-molded patterns under surface stiffening elicited by shear rigidity functionalization.

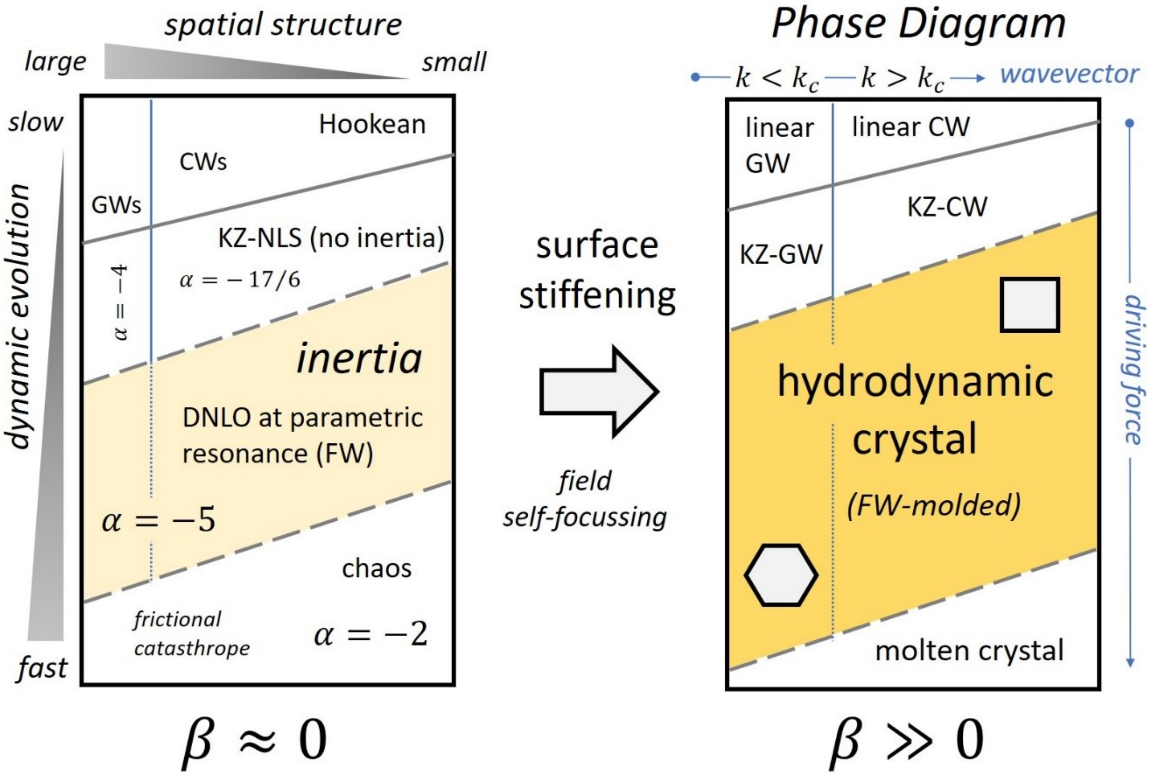

Supplementary Figure N10. Dynamic NLSW states under monochromatic liquid vibration. A) Spectral signatures as referred to the DNLO model in the absence of wavefield self-interaction ($\beta \approx 0$). The different NLSW states are classified in terms of the evolution of their dynamic organization and the spatial structure as endowed by wave dispersion. B) Phase diagram at the disorder-order transition induced by surface stiffening ($\beta > 0$). The appearance of hydrodynamic crystals is a natural consequence of stiffening-induced Faraday wavefield self-focusing causing the strong wave phase locking observed in experiments; FW-molded patterns (see Figure 4 in the main text).

3-2. Hydrodynamic crystalline state: FW-freezing under surface stiffening

As a first cause for the FW-distinctive spectral slope ($\alpha = -5$), bulk inertia has been revealed, together with a surface response self-focused under shear stiffening, as the crucial ingredients at the genesis of the new phase of hydrodynamic crystals identified in the experiments. The bulk inertia sets up for repeating back and forth nonlinear harmonic and parametrically amplified subharmonic resonances sustained indefinitely by the external force in the practical absence of frictional losses (see Supplementary

Note 1). Therefore, we consider the bulk inertia imposed by the vibrating liquid as a mandatory requirement for FW-formation, thus the indispensable material component at the core of the fundamental interactions underlying crystal genesis. The intrinsic details of the FW response have been coarse-grained behind the DNLO characteristics (without explicit account of the spatial structure connecting different wavefield emplacements), thus, the predictive value of the DNLO-MMT framework is limited, just qualitative at the effects of explaining the universalizing transition to the FW regime dominated by bulk inertia. At the present state of work within the scope of this paper, DNLO-MMT constitutes just a depiction of reality at a single surface emplacement (one dimensional), although with a potential to describe the spatial extension of the 2D-wavefield using the spatial operators of symmetry encoded in the MMT program [33,38].

3-3. DNLO-NLS equivalent scalar wavefield: wave equation

The essential features of hydrodynamic skeletonization piloting FW-genesis have been captured under a one-dimensional DNLO-like dynamics, Eq. (1). A more complete theory might include a program for spatial structure as generalized in the MMT model. This has been discussed to exist under the minimal NLS universality class of wave diffusion representing the dispersive wave structure encoded in the hydrodynamically disordered states previous to FW formation (see Supplementary Figure N9); it corresponds to MMT($\mu = 2$; $\nu = 0$) in Eq. (26), which give rise to the Fickian-like NLS structure in Eq. (28). Let's first built the linear conservative equations for the surface wavefield with the same spatial structure as the NLS equation (diffusive-like). In Supplementary Figure N11, we consider the surface as an elastic string made of N springs of rigidity k, size l and mass m spaced over the whole length of the surface $L = Nl$, and total mass $M = Nm$. For a one-dimensional array of coupled oscillators as cartooned in Supplementary Figure N11, the wave equation can be derived in the same way as the transverse waves in a tensioned string [39]; ignoring dissipation, once inertia is accounted for, one gets:

$$\frac{\partial^2 \psi}{\partial t^2} - c^2 \frac{\partial^2 \psi}{\partial x^2} = F(t)/m \quad (29)$$

which describes a forced, spatially structured wavefield with a velocity of propagation as given by the natural frequency of the composing harmonic oscillators $c^2 \equiv kl^2/m \equiv$

KL^2/M (with $K \equiv k/N$ being the spring constant of the elastic array composed by individual springs as coupled in parallel; see Supplementary Figure N11).

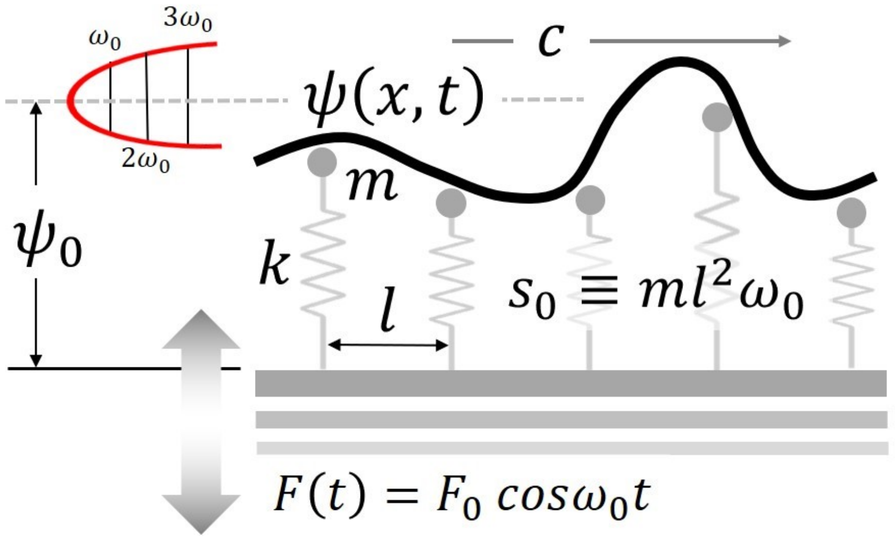

Supplementary Figure N11. Discretized scalar wavefield under monochromatic resonant coupling in a tensioned surface with lateral structure. The wavefield $\psi(x,t)$ varies as a function of the spatial coordinates and time as due to the resonant response of an array of oscillators coupled in parallel against the driving action of an external monochromatic source $F(t) = F_0\, cos\omega_0 t.$ Oscillator characteristics: spring constant (k), mass (m), characteristic lateral length (l). When the liquid is vibrated the normal modes are excited at discretized frequencies $\omega_n = n\omega_0$, which correspond to integer multiples of an elemental unit of action; in terms of systemic fundamental properties of mass, space and time: $s_0 \equiv ml^2\omega_0$, or $s_0 = F_0 l/\omega_0$ in terms of the applied force F_0, which determines the apparent inertial mass involved in the interaction as $m \equiv F_0/l\omega_0^2$. The wavefield propagates at velocities $c = l\omega_0$. Because motion is forced at the single driving frequency ω_0, all the systemic quantities are essentially discretized, so that waving responses appear naturally quantized in the classical sense of the term as a systemic discretization into single levels of energy $\omega_n = n\omega_0$, acted by an action s_n determined by integers $n = s_n/s_0$ of the elemental quantum of action s_0 (see the discretized energy diagram as a potential well in red).

3-4. Wavefield discretization: elemental quantum of action

The equivalent string response appears classically quantized in the given sense that discrete modes are driven by the single frequency ω_0, and the wave velocities are intrinsically selected by discretized pieces of length l and period $2\pi/\omega_0$. Because the term $\omega_0^2 = k/m$, corresponds to the natural response of the composing springs, then $c = l\omega_0$ in view of Eq. (29) (with l being an elemental length). Therefore, an equivalent "quantum" of systemic action can be defined in terms of elemental momentum and length as $s_0 \equiv pl$; since $p = mc$, the systemic unit of action is $s_0 \equiv mcl$, in terms of the wave velocity $c = l\omega_0$, which is discrete in own nature. The inertial mass describes the effective mass that provides systemic momentum to the surface due to resonant forcing from the bulk, this is $m \equiv F_0/l\omega_0^2$ (see Supplementary Figure N11). Looking

back at Eq. (29), the case $F_0 = 0$ (thus $m = 0$) represents a discretized linear wavefield for an unforced string in a massless oscillator (if seen the leftmost part of this equation). Equivalently, this equation represents the motion of a free particle (if looking to the right, i.e. $F/m = 0$). The forced wave equation can be interpreted in this way, as the wavefield created by a forced mass-spring system. By introducing action to the system ($F_0 \neq 0$), the equation of motion corresponds to an oscillating string intercoupled with a moving particle of inertial mass $m \equiv F_0/l\omega_0^2$, which drives the wavefield at resonance with the forcing $F(t)$ (the rightmost part of Eq. (29) is seen as a source for action). Assuming Hookean causality between generating force and generated wavefield, i.e. $F(t) = -k\psi(t)$, the forcing term can be expressed in terms of the elemental action s_0 of a fictitious particle of mass m as $F(t)/m = -(m^2c^4/s_0^2)\psi(t)$; consequently, Eq. (29) can be reorganized as follows:

$$\frac{\partial^2\psi}{\partial t^2} - c^2\frac{\partial^2\psi}{\partial x^2} + \frac{m^2c^4}{s_0^2}\psi = 0 \qquad (30)$$

which takes the same form as the Klein-Gordon (KG) equation for the quantized version of the relativistic energy-momentum relation [40]. The action appears here “quantized”, or discretized in units of s_0, corresponding to the “quantum of action” for the mass-spring equivalent particle that generates the field; in terms of the elemental pieces of mass, length and time of the system, the quantum writes:

$$s_0 \equiv ml^2\omega_0 \qquad (31)$$

Since m, l and ω_0^{-1} are discrete in their own definition, this fundamental unit of systemic action defined is essentially discrete in its own nature; i.e. s_0 can be interpreted as the elemental (and minimal) unit of action the system uses to get moving. Thus, the quantum of action s_0 is introduced as a systemic discretization in the classical sense of the term (not as the Planck constant appeared in quantum mechanics as a fundamental ground for the minimal quantity of electromagnetic action as measurable under the uncertainty principle).

3-5. Linear Lagrangian: effective mass interaction

Since each term in Eq. (30) is linear in the field ψ, the forced wave equation represents a wavefield under discretized action from a quadratic Lagrangian density:

$$\mathcal{L}_2(x,t) \equiv \mathcal{T} - \mathcal{U}_2 = \frac{1}{2}(\partial_t\psi)^2 - \frac{c^2}{2}(\partial_x\psi)^2 - \frac{m^2c^4}{s_0^2}\psi^2 \quad (32)$$

which is composed by kinetic energy $\mathcal{T}$ (the wave action) and two potential terms appearing in $\mathcal{U}_2$. The first one, of "quantized" strength $c^2 = l^2\omega_0^2$, is the Hookean field on the surface strain ($c^2 = kl^2/m$). The term proportional to m^2 is known as a mass term, or inertial potential due to its subsequent interpretation in terms of particle mass. This quadratic mass term is now at the core of the interaction as the first cause for inertial resonance (as in the DNLO theory; see Supplementary Note 1).

The linear Lagrangian with the KG-structure can be interpreted as an effective field equation for the discrete wave-particle oscillations ψ_n created upon monochromatic excitation. Because the quantum of action is fixed by the driving frequency $s_0 \equiv ml^2\omega_0$, the harmonic frequencies correspond to multiple integers of this quantum, i.e. $\omega_n = n\omega_0 = ns_0/ml^2$. This naturally discretized response has been cartooned in Supplementary Figure N11, which brings to light why only discrete states are available from the elemental quanta of action s_0. So that responding linearly at the natural frequency ω_0 requires the system with an action s_0. A two-folded action $s_2 = 2s_0$ is required for a nonlinear response at the first harmonics $\omega_2 = 2\omega_0$, $s_3 = 3s_0$ for $\omega_3 = 3\omega_0$, … and so on, up to the highest level $\omega_{max} = n_{max}\omega_0$. The discrete solutions are obtained as a quantized classical field $\psi = \{\psi_n\}$, whose discretized quanta at the harmonic frequencies ω_n, are system-representing quasi-particles with an effective potential mass m considered as the wavefield generator for given driving strength and frequency, this is $m \equiv F_0/l\omega_0^2$.

3-6. Accounting for DNLO-stiffening: quartic self-interaction

In order to account for nonlinear Duffing-like stiffening interaction, the quadratic Lagrangian can be complemented with a scalar potential for the quartic self-interaction $\mathcal{U}_4 = g_4\psi^4/4$. in terms of the nonlinear Lagrangian density, $\mathcal{L}_4 = \mathcal{L}_2 - \mathcal{U}_4$, the global action for the discretized DNLO field can be written as:

$$S = \int \mathcal{L}_4(x,t)\, dx\, dt \quad (33)$$

from which the equation of motion is obtained as the Euler-Lagrange minimizer:

$$\frac{\partial^2\psi}{\partial t^2} - c^2\frac{\partial^2\psi}{\partial x^2} + \frac{m^2c^4}{s_0^2}\psi + g_4\psi^3 = 0 \quad (34)$$

with nonlinear strengthening proportional to the Duffing parameter ($g_4 \sim \beta\omega_0^2$), which is also subjected to discretization imposed by the driving frequency.

More synthetically, we have constructed a scalar field theory for a nonlinear (DNLO-like) self-interaction in addition to a mass term. They both recapitulate the generating role of inertia and wavefield self-focusing as the fundamental components at the core of a scalar potential, which defines the Lagrangian connection between potential energy and momentum in a system-equivalent quasiparticle at wavefield interaction (see Lifshitz's field below). As an outlook on the future development of this novel physics, we have preliminarily formulated the essentials of the classical field theory the undergrounds the new material object here discovered, the hydrodynamic 2D-crystal. Aligned with previous arguments and evidences on the analogy of Faraday waves and De Broglie matter waves [41,42], we are further explaining how the physics here described could represent a unique realization of a classical matter wave able to freeze a crystalline pattern in two dimensions.

3-7. Faraday matter waves

The inertia-generated FWs exhibit a material nature as a momentum equivalent in the moving liquid surface at parametric resonance with the bulk fluid: the DNLO-model has shown the parametric FW's supported in the subharmonic wavefield as generated in an elastic body (the fluid surface), at resonance with a material content that endows strong inertia (the bulky fluid). Both entities exhibit an intrinsic wave-particle duality. Such reciprocity can be stated as matter waves in an analogous formulation as De Broglie duality, but in the same classical sense stated above. Because we have encoded the discretized structure of the normal DNLO-modes of a structured surface as an infinity of coupled oscillators that represent its elastic response, the KG equation obeys the particle-wavefield dispersion relationship; from Eq. (34) in the linear case ($g_4 = 0$), through of the Fourier decomposition of the wavefield $\psi = \psi_0 e^{i(kx-\omega t)}$, one gets the DNLO-dispersion equation:

$$\omega_k^2 = k^2 c^2 + \frac{m^2 c^4}{s_0^2} \qquad (35)$$

which describes the systemic spectral blue-shift of the massive system with respect to the natural frequency; for the massless oscillator ($m = 0$), the non-dispersive harmonic oscillator is recovered as $\omega = kc$.

By expressing Eq. (35) in terms of the linear momentum $p = mc$, the quantized energy-momentum relation for the DNLO is:

$$\frac{\omega_k^2}{c^2} - k^2 = \frac{p^2}{s_0^2} \qquad (36)$$

which can be interpreted as the wave-particle duality relationship for the linear wavefield of matter waves discretized by the elemental quantum of action $s_0 \equiv ml^2\omega_0$. For a matter DNLO-like wave ($p \neq 0$), wave characteristics are completely discretized in terms of this classically unit (quantum) of action; two limits are analysed:

a) Low wavevectors *($k \to 0$),* representing surface oscillations of large wavelength ($\lambda = 2\pi/k \to \infty$); since $c = \omega_k/k = \omega\lambda/2\pi$, we obtain in this case the classical analogous to the quantum wave-particle De Broglie relationship:

$$\lambda_0 \approx \frac{2\pi s_0}{p} \qquad (37)$$

b) High wavevectors *($k \to \infty$),* representing surface oscillations of short wavelength ($\lambda = 2\pi/k \to 0$); in this case, one gets:

$$\lambda_\infty \approx i\,\frac{2\pi S_0}{p} \qquad (38)$$

which represents an amplified wave with an exponential growth. Note that these unstable high-k modes would be killed by friction.

3-8. Self-focused wavefield spatial structure: Lifshitz's field for Faraday waves

Under the inertial resonant conditions captured by the nonlinear KG-equation at high enough self-focusing (induced e.g. under the surface "stiffening" endowed by the adsorbed layer of aescin), the Faraday wavefield should be prone to condense with a spatial organization mastered by the mother wavelength $\lambda_{1/2}$, which is inversely proportional to a piloting subharmonic frequency $\omega_{1/2} = \omega_0/2$. The discrete FWs organize in patterns of this mastering mode, which dimensionally divide the surface in organized spaces, or unit cells, as equal intervals of alternating reciprocating forces dynamically balanced against each other. These unitary cells should maintain the

properties of periodicity and symmetry of the master mode across every possible dimension of space and time. The discrete couplings between successive harmonics do persist as indirectly fixed by the driving source at parametric resonance (see Supplementary Note 1). Frictional losses are minimal within this subharmonic channel as it corresponds to a subsidiary wavefield amplified under parametric resonance at dynamic evolution characterized by a reduced damping. From the discretized KG-perspective above depicted within the MMT-DNLO dynamical framework, we have envisaged a minimal program in scalar field theory that captures the essential ingredients of wave inertia and self-focused wave dispersion at the core of the spatiotemporal coherence leading to FW formation. The FW modes of vibration restored by surface changes of area (capillary regime) should satisfy a wave equation with the conventional harmonic form; in the absence of dissipation:

$$m\partial_{tt}\psi = -\kappa(-\Delta)^{\mu}\psi \tag{39}$$

with m being the inertial mass of the Faraday wave at rest, and κ a generalized spring constant with the units $[\kappa] = M \cdot L^{2\mu} \cdot T^{-2}$ ($\Delta = \partial_{xx}$ is the Laplacian operator). The MMT-exponent μ recapitulates the spatial structure of the FW field; if Fourier-synthetized in space domain:

$$\psi(x,t) = \int \psi_k(t)e^{ikx}dk \tag{40}$$

one expects harmonic solutions as:

$$\partial_{tt}\psi_k = -\omega_k^2\psi_k \tag{41}$$

with dispersion law giving the natural frequency of the mode k as:

$$\omega_k = (\kappa/m)^{1/2}k^{\mu} \tag{42}$$

By adopting the field theory perspective, these behavior can be described by a Lagrangian density of the Lifshitz class for a FW-equivalent particle of inertial mass m taking part of the self-interacting field characterized by the spatial structure operator $(-\Delta)^{\mu}$ [43]; using the KG-formulation in reduced units ($c = 1$, $s_0 = 1$):

$$\mathcal{L} = \frac{1}{2}[(\partial_t\psi)^2 - \psi(m^2 + (-\Delta)^{\mu})\psi] \tag{43}$$

Through of the exponent $\mu > 0$, the Lagrangian in Eq. (43) contains self-interaction terms at higher order derivatives than the pure harmonic oscillator without spatial structure ($\mu = 0$). Consequently, the equation of motion can be expressed as:

$$\partial_{tt}\psi_k = -\Omega_k^2\psi_k \qquad (44)$$

with an effective natural frequency:

$$\Omega_k^2 = m^2 + k^{2\mu} \qquad (45)$$

where the MMT-exponent μ could recapitulate the level of wavefield self-interaction in terms of stiffness as a breakdown of the discretized symmetry of the harmonic Hamiltonian. The case $\mu = 1$ describes the well-known fields at unitary self-interaction, mass m and diffusive-like wave dispersion $\omega_k = k^2$.

The Lifshitz's action generator in Eq. (43) is envisaged as a minimal kernel for the development of a forthcoming theory of hydrodynamic crystals as frozen Faraday waves under a high amount of surface stiffness. This theory is sufficiently comprehensive of the essential material contents, i.e. bulky inertial mass and surface elastic response both discretized upon the monochromatic forcing.

Supplementary Note 4

Synthesis of aqueous suspensions of microparticles

PS-MAA particle suspension.

Aqueous suspensions of spherical particles of poly(styrene-co-methacrylic acid) (PS-MAA) were prepared from surfactant-free emulsion by a conventional polymerization process of both monomers [44]. The polymerization reaction was carried out in a 500 mL round-bottomed, five-necked flask. In the flask outlets, a water-cooled reflux condenser, a T-shaped stirrer, a gas inlet and a contact thermometer were fitted. One outlet was used for introducing the chemicals of the reaction. The flask was introduced in a thermostat water bath which controls the reaction temperature to ± 2℃. The synthetic process starts by introducing 253 mL of water, 18 g of styrene and 1.58 g of methacrylic acid. The styrene was washed with a NaOH 0.1M water solution to remove the monomer inhibitors. The stirring was fitted to 350 rpm and nitrogen gas was bubble during all reaction time to remove any oxygen traces. When the temperature arrived to 85 ºC, 0.22 g of potassium persulfate (KPS) was added as initiator of the

polymerization reaction. After eight hours of reaction, the flask is removed from the bath and cooled in a water-ice mixture. The resulting polymer suspension is dialyzed against water over three weeks.

Scanning electron microscopy (SEM) was performed with a JEOL-JSM-6330F electron microscope operating at 10kV. By analysing SEM-images using ImageJ, the average diameter was estimated into 190 nm. Measurements of dynamic light scattering (DLS) with a 90-Plus/BI-MAS apparatus was performed to characterize particle size distribution. An average particle diameter of 200 nm was obtained, with a relative standard deviation of 0.030 indicating an extremely low size polydispersity. Electrophoretic mobilities were estimated by using the ZETA-PALS mode of the BI-MAS equipment. The measures were performed at room temperature and an ionic strength of 1mM of KNO_3. The analysis of the data gave a zeta-potential of 47.3 mV on negative charged particles, with charges come from the persulfate fragments.

PS particle suspension

We also prepared Polystyrene (PS) microparticles by following the same synthetic route as with PS-MMA, without adding MAA monomer and introducing a small monomer charge of the reactor (9 g). The slow polymerization rate of styrene required to perform the polymerization during 24h.

A dilute PS suspension was measured by DLS and the average particle diameter was 313 nm, with a relative standard deviation of 0,037.

Supplementary Note 5

Dispersion relation of GC-waves in the presence of a surface viscoelastic film

We are interested in the correction to GC-wave dispersion due to surface elasticity arising from a viscoelastic film adsorbed to the surface. The classical treatment of Hansen and Mann (HM) considers linear hydrodynamics for the propagation of capillary waves on viscoelastic surfaces [45]. To account for the case of surface waves propagating on aescin covered surfaces as the data represented in the Fig. 3b) of the main text, we considered the HM-theory for a highly rigid surface film, this is at the

material limit $\xi = G/\sigma \gg 1$. Essentially, the viscoelastic film is known to affect capillary motions inducing weak changes on the propagation characteristics (ω and k) companioned by strong wave damping (β). For the propagation frequency of the capillary waves, the HM-theory predicts a non-monotonic variation with the capillary wavenumber, from the ideal Kelvin value corresponding to the bare surface, this is $\omega_0 = (\sigma k^3/\rho)^{1/2}$ for $\xi = 0$, through an intermediate maximum of optimal propagation rate that is dependent on the elasticity ratio $\xi = G/\sigma$, down to the limit $\xi \to \infty$ ($G \gg \sigma$), where the propagation frequency reaches a high rigidity value $\omega_\infty = \sigma k/\eta$, which is completely independent of ξ but compatible with the overdamped solution for a capillary ripple in the bare surface of a liquid of viscosity η. Because aescin monolayers adsorbed at concentrations close to cmc are terribly rigid ($\xi \approx 30$, since $G \approx 1\,N/m$ and $\sigma \approx 0.03\,N/m$) as compared to typical adsorbed films of typical surfactants $\xi \ll 1$, we considered the HM-picture of a highly viscoelastic film to describe the propagation characteristics of the surface waves in the presence of a rigid monolayer of aescin adsorbed at the free surface of water. Briefly, we first took the ideal solution for GC-waves considered in Methods, and then reorganized it with the reduced form considered in HM's work:

$$y(\omega) = \frac{\rho\omega^2}{\rho g k + \sigma k^3} \quad (46)$$

Because the perturbative corrections to the ideal solution $y(\omega_{GC}) = 1$ in the HM-theory can be expressed in terms of the elasticity ratio $\xi = G/\sigma$ and the dimensionless factor $u = \eta\omega/\sigma k$, further elaborating on the result for a highly viscoelastic film (Eq. 38 in the 1964's paper), in the limit $\xi \to \infty$ we obtained the approximate formula:

$$y_{\xi\to\infty}(\omega) \approx 1 - \left(\frac{\omega}{2\omega_\infty}\right)^{1/2} + \frac{1}{4}\frac{\omega}{\omega_\infty} \quad (47)$$

where we have substituted the factor $u = \eta\omega/\sigma k = \omega/\omega_\infty$ in the HM-theory as written in terms of the overdamped frequency $\omega_\infty = \sigma k/\eta$. This equation represents the high-rigidity perturbation limit to the GCW propagation in a free surface covered with a viscoelastic film. It was numerically resolved to obtain the limiting solution $\lambda_{\xi\to\infty}(\omega)$ plotted in Fig. 3b).

Supplementary Note 6

Experimental setup for the interfacial rheology measurement

We used a commercial hybrid rheometer (HDR100, TA Instruments) working in the surface shear mode for measuring the viscoelastic modulus of surface films at the air/water interface. The rationale is explained in Supplementary Figure N12.

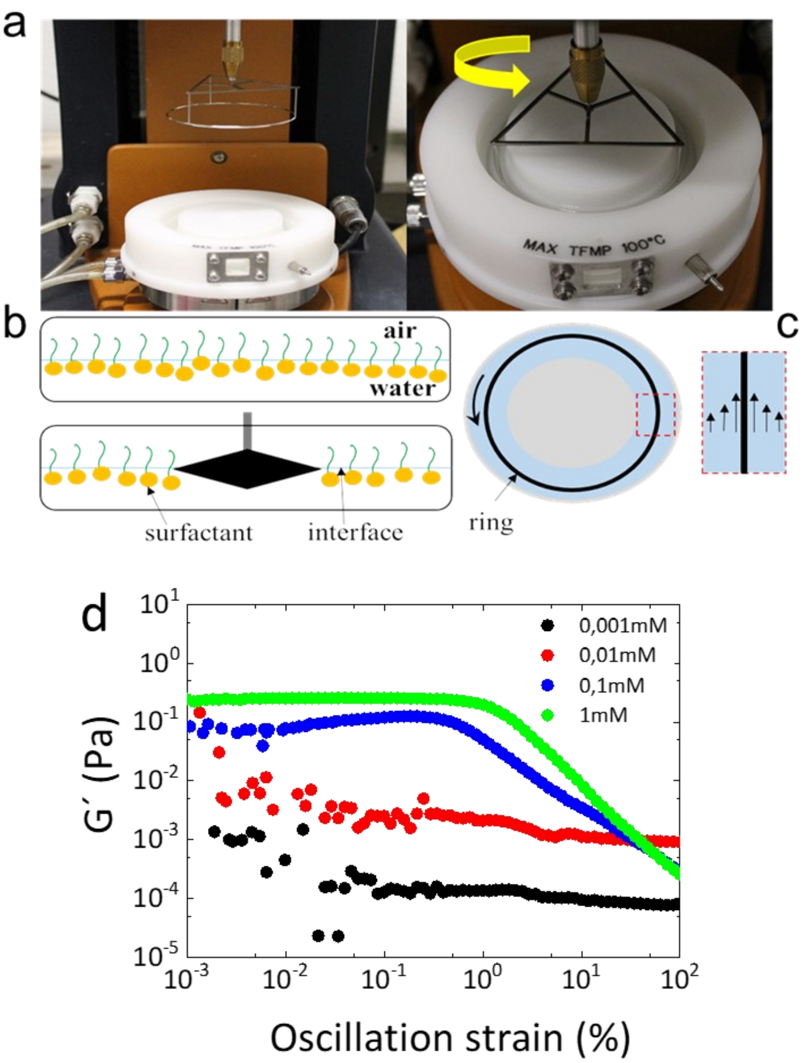

Supplementary Figure N12. a) Du Nöuy ring configuration with the thermostatic vessel. b) The ring is in contact with the aqueous solution surface with surfactant, which develops a surface monolayer (here simplified by drawing a schematic head-tail amphiphilic molecule configuration). c) The rheological measurement is carried out by in-plane shear force stressed on the surface monolayer by the ring tool. d) Experimental measurements of the stress-strain relationship in the same systems considered in this work. Data in the Figure 5 of the main text were measured at 0.1% strain, corresponding to the linear regime.

Supplementary References

[1] O. M. Phillips. Wave interactions - the evolution of an idea. J. Fluid. Mech. 106, 215 (1981).

[2] L.W. Schwartz & J.D. Fenton. Strongly nonlinear waves. Annu. Rev. Fluid Mech. 14, 39 (1982).

[3] V.E. Zakharov, V.S. L'vov & G. Falkovich. *Kolmogorov Spectra of Turbulence I: Wave Turbulence* (Springer-Verlag, 1992).

[4] E. Kartashova. Exact and quasiresonances in discrete water wave turbulence. Phys. Rev. Lett. 98, 214502 (2007).

[5] J. L. Hammack and D. M. Henderson, Resonant interactions among surface water waves. Annu. Rev. Fluid Mech. 25: 55 (1993).

[6] M. Faraday. On a peculiar class of acoustical figures and on certain forms assumed by a group of particles upon vibrating elastic surfaces. Phil. Trans. R. Soc. London 121, 299 (1831); ibid. On the forms and states of fluids on vibrating elastic surfaces. Philos. Trans. 121, 319 (1831).

[7] J. Miles and D. Henderson. Parametrically forced surface waves. Annu. Rev. Fluid Mech. 22, 143 (1990).

[8] G. B. Whitham, Linear and Nonlinear Waves (Wiley Series in Pure and Applied Faraday Mathematics, 1999).

[9] S. Nazarenko. Wave Turbulence (Springer, 2011).

[10] V.E. Zakharov. Stability of periodic waves of finite amplitude on the surface of a deep fluid. Zhurnal Prildadnoi Mekhaniki i Tekhnicheskoi Fiziki, 9, 86 (1968).

[11] G. Falkovich, K. Gawedzky and M. Vergassola. Particles and fields in fluid turbulence. Re. Mod. Phys. 73, 913 (2001).

[12] S. Nazarenko and S. Lukaschuk, Wave turbulence on water surface, Annu. Rev. Condens. Matter Phys. 7, 61 (2016).

[13] T.B. Benjamin and U. Ursell. The stability of the plane free surface of a liquid in vertical periodic motion. Proc. R. Soc. Lond. A 225, 505 (1954).

[14] K. Kumar and L. S. Tuckerman, Parametric instability of the interface between two fluids, J. Fluid Mech. 279, 49 (1994).

[15] N. Minorsky, Nonlinear Oscillations (Robert E. Krieger, New York, 1974).

[16] 5. Nayfeh, A. & Mook, D. Nonlinear Oscillations (Wiley-Interscience, New York, 1979).

[17] T.I. Fossen and H. Nijmeijer, eds. Parametric Resonance in Dynamical Systems (Springer, 2012). ISBN 978-1-4614-1042-3

[18] J. Bechhoefer and B. Johnson. A simple model for Faraday waves (1996).

[19] Y. Ueda. Randomly transitional phenomena in the system governed by Duffing's equation. J. Stat. Phys. 20, 181 (1979).

[20] Polytec® PDV-100 Portable Digital Vibrometer: technical document at OM_DS_PDV-100_E_42474.pdf (polytec.com)

[21] Wolfram Mathematica v12.0: https://www.wolfram.com/mathematica/?source=nav

[22] G.B. Arfken & H.J. Weber. *Mathematical Methods for Physicists* (Harcourt, San Diego, 2001).

[23] O.M. Phillips. On the generation of waves by turbulent wind. J. Fluid Mech. 2, 417 (1957).

[24] O.M. Phillips. The equilibrium range in the spectrum of wind-generated waves. J. Fluid Mech. 4, 426 (1958).

[25] W.B. Wright, R. Budakian, D.J. Pine and S.J. Putterman. Imaging of intermittency in ripple-wave turbulence. Science 278, 1609 (1997).

[26] D. Snouck, M.T. Westra and W. van de Water. Turbulent parametric surface waves. Phys. Fluids 21, 025102 (2009).

[27] H. Punzman, M.G. Shats and H. Xia. Phase randomization of three-wave interactions in capillary waves. Phys. Rev. Lett. 103, 064502 (2009).

[28] S. Novak and R.G. Frehlich, Transition to chaos in the Duffing oscillator. Phys Rev A 26, 3660 (1982).

[29] L.D. Landau, On the problem of turbulence, C.R. Acad. Sci. URSS, 44, 311 (1944).

[30] Collected Papers of L.D. Landau, D. Ter Haar, ed. (1st Edition, Pergamon, 1965) ISBN: 9781483152707.

[31] V.E. Zakharov. The algebra of integrals of motion of two-dimensional hydrodynamics in Clebsch variables. Moscow Institute of Electronic Machinery Construction, UDC 517.9, pp. 189-196 (Plenum, 1990). Translated from Funktsional'nyi Analiz i Ego Prilozheniya, Vol. 23, No. 3, pp. 32-44 (1989).

[32] L.D. Landau and E.M. Lifshitz. Fluid Mechanics, 2nd Ed. (Butterworth-Heinemann, 1987).

[33] A.J. Majda, D.W. McLaughlin and E. G. Tabak. A one-dimensional model for dispersive wave turbulence. J. Nonlinear Sci. 6, 9 (1997).

[34] V. Zakharov, P. Guyenne, A.N. Pushkarev, F. Dias, Wave turbulence in one-dimensional models, Physica D 152–153, 573 (2001).

[35] V. Zakharov, F. Dias, A. Pushkarev, One-dimensional wave turbulence, Phys. Rep. 398, 1 (2004).

[36] A. Pushkarev, On the Kolmogorov and frozen turbulence in numerical simulation of capillary waves, Eur. J. Mech. B/Fluids 18, 345 (1999).

[37] D. Andelman and R. E. Rosensweig, Modulated phases: review and recent results, *J. Phys. Chem. B* 113, 3785 (2009).

[38] S. Chibbaro, F. De Lillo, M. Onorato, Weak versus strong wave turbulence in the MMT model, Phys. Rev. Fluids 2, 052603 (2017).

[39] H. Goldstein, Classical Mechanics, 2nd Ed. (Addison-Wesley, 1980).

[40] H. Feshbach and F. Villars. Elementary relativistic wave mechanics of spin 0 and spin 1/2 particles. *Rev. Mod. Phys*. 30, 24 (1958).

[41] Y. Couder, S. Protière, E. Fort and A. Boudaoud. Walking and orbiting droplets. Nature 437, 208 (2005).

[42] J.W.M. Bush, Quantum mechanics writ large, Proc. Nat. Acad. Sci. USA, 107, 17455 (2010).

[43] L.D. Landau and E.M. Lifshitz. Classical Theory of Fields, 3rd ed. (Pergamon, London 1971).

[44] M. C. Carbajo, E. Climent, E. Enciso and M.J. Torralvo. Characterization of latex particles arrays by gas adsorption. J. Coll. Interface Sci., 284, 639 (2005).

[45] R. S. Hansen and J.A. Mann. Propagation characteristics of capillary ripples. I. The theory of velocity dispersion and amplitude attenuation of plane capillary waves on viscoelastic films. J. Appl. Phys. 35, 152 (1964).
